# Socioeconomic, demographic and geographic disparities in accessibility to food pantries in the united States

**DOI:** 10.1038/s41598-026-35784-z

**Published:** 2026-01-26

**Authors:** Yifan Zhang, Minhwa Lee, Jason B. Gibbons, Huan-Yuan Chen, Yan Wang, Zonghai Yao, Olivia Bennett, Feiyun Ouyang, David Levy, Katherine L. Tucker, Hong Yu

**Affiliations:** 1 Miner School of Computer & Information Science, University of Massachusetts Lowell, Lowell, MA USA; 2 Division of Pharmacoepidemiology and Pharmacoeconomics, Brigham and Women’s Hospital, Harvard Medical School, Boston, MA USA; 3 Robert and Donna Manning College of Information and Computer Sciences, University of Massachusetts Amherst, Amherst, MA USA; 4 Department of Psychology, University of Massachusetts Lowell, Lowell, MA USA; 5 Department of Economics, Brandeis University, Waltham, MA USA; 6 Center of Biomedical and Health Research in Data Sciences, University of Massachusetts Lowell, Lowell, MA USA; 7 Department of Biomedical & Nutritional Sciences and Center for Population Health, University of Massachusetts Lowell, Lowell, MA USA; 8 Center for Healthcare Organization and Implementation Research, VA Bedford Health Care System, Bedford, MA USA

**Keywords:** Risk factors, Public health

## Abstract

Improving access to food pantries (FPs) may help address food insecurity, yet national-level assessments of geographic and socioeconomic disparities remain limited. We created a dataset of 34,475 FPs across all 50 states and the District of Columbia and analyzed their accessibility to the nearest Census block group (BG). We defined accessibility as high, medium, or low based on travel time or distance. Analyses of 239,780 BGs showed that 23.4% had low FP access. We identified geographic disparities across regions and states. For example, states in the Northeast generally have higher FP accessibility, while those in the South show lower access. Although rural BGs are more socioeconomically disadvantaged than urban ones (mean ADI: 71.3 vs. 45.8), they generally have better FP access (only 8.5% with low access vs. 26.5% in urban areas). However, some rural BGs with low FP accessibility tend to have high ADI values. In urban areas, FP placement shows relatively good alignment withneighborhood needs based on analyses of socioeconomic characteristics. In contrast, rural access patterns are more mixed. For example, less-educated rural populations face greater barriers. Findings from this study can inform policy and implementation strategies to improve FP access in underserved communities.

**Supplementary Information:**

The online version contains supplementary material available at 10.1038/s41598-026-35784-z.

## Introduction

In 2022, 44.2 million Americans lived in food-insecure households, a 30% increase over the previous year^1^. Food insecurity, a condition in which households lack access to adequate food because of limited money or other resources, is a leading health and nutrition issue in the United States^2^. It is associated with numerous adverse health outcomes, including increased risk of chronic diseases such as diabetes, mental health problems including depression and anxiety, and disordered eating behaviors^3–7^. While federal programs like the Supplemental Nutrition Assistance Program (SNAP), Women, Infants, and Children (WIC), and The Emergency Food Assistance Program (TEFAP) offer support to low-income individuals and at-risk women and children^8,9^, eligibility requirements can exclude many in need. For example, 37% of individuals experiencing food insecurity do not qualify for SNAP due to income thresholds, which ranged from 130 to 200% of the federal poverty line, equivalent to an annual income between $39,000 and $60,000 for a family of four as of January 2023^10^. Further, existing programs often fail to provide sufficient food to fully meet the needs of food-insecure households^11^.

Food pantries (FPs) play a critical role in supplementing federal programs. Operated primarily by nonprofit or faith-based organizations, FPs provide direct food access and typically do not impose strict income requirements^12^, making them accessible to households who fall through eligibility gaps. Studies have shown that FPs help mitigate food insecurity at the community level^13^, and that pantry-based interventions, such as nutrition education, client-choice interventions, and enhanced food displays, have been shown to improve clients’ diet-related outcomes^14^. Taken together, FPs constitute a major component of the charitable food system, serving over 50 million people in the United States^15^.

FPs often work with state, tribal, and local health and safety agencies that oversee the appropriate food provision to the neighborhoods within their service areas^16^. Geographic accessibility (hereafter referred to as *accessibility*) is an important consideration in the establishment of FPs. It is usually defined as the ease of getting to the FP^17,18^. Ideally, the locations of FPs should be proximal to the food-insecure population, targeting neighborhoods with high socioeconomic vulnerability^19–21^. These include populations that are disproportionately affected by food insecurity, such as low-income households, Black/African American and Hispanic households, children, immigrant communities, residents of certain geographic areas (e.g., rural regions, urban centers, the South) and single-parent households^22–25^.

Most existing studies measuring access to FPs have been mainly focused on specific counties or cities within a single U.S. state^16,19,26–32^. For example, a study conducted in Santa Clara County, California, found that food assistance distribution sites are generally near communities experiencing high levels of poverty and food insecurity^33^. Another study in El Paso County, Texas, reported that neighborhoods characterized by socioeconomic disadvantage, household instability, disability, minority status, language barriers, and greater proximity to emergency food sites exhibited significantly higher levels of both food transfer coverage and intensity^34^. Caspi et al. found that census tracts with a greater concentration of racial/ethnic minority populations and some foreign-born groups (i.e., East African, Latin American, and Southeast Asian) are more likely to be located near food shelves in Twin Cities, Minnesota^21^. While prior studies have provided valuable insights, understanding of FP accessibility for socioeconomically and geographically vulnerable populations at the national level remains limited.

One national study found that over 40% high-poverty neighborhoods without a supermarket had the access to emergency food pantry service^20^. However, the scope of this analysis was restricted to the census tract level and included only descriptive analysis. In contrast, our study conducts a nationwide analysis using census block groups (BGs), which are small geographic groupings that contain between 600 to 3,000 individuals who are more socio-demographically homogeneous than at the tract or county levels. BGs have been shown to be superior to other geographic entities (e.g., Zip codes^35^ and census tract data^36^) for public health research. Moreover, whereas the national study identified the presence of FPs, it did not quantify accessibility. In contrast, our study defines accessibility based on travel time or distance. We integrate descriptive statistics with univariate regression models to comprehensively examine FP accessibility in the context of socioeconomic, demographic, and geographic disparities.

Specifically, we evaluated differences in access to FPs at the national level based on geographic location and demographic and socioeconomic characteristics. To achieve this aim, we focused on the following objectives: (1) creating a comprehensive national dataset of FPs and their geolocation information; (2) determining associations between FP accessibility and socioeconomic vulnerability using a combination of univariate analyses and regression models; and (3) evaluating whether socioeconomically vulnerable neighborhoods in urban areas can reach their nearest FP within a reasonable time by walking or public transit, and whether those in rural areas can do so within a reasonable driving distance. We categorized 239,780 BGs as rural or urban across all 50 states and the District of Columbia and conducted separate analyses for each category. Additionally, we assessed the socioeconomic vulnerability of each BG using the Area Deprivation Index (ADI), where the most socioeconomically disadvantaged BG has an ADI of 100^37,38^. This research provides a detailed examination of FP access disparities, offering comparative analyses within and across both urban and rural areas.

## Results

### FP dataset validation: precision and recall assessment

We created a national dataset of 34,475 FPs. The dataset was evaluated for precision and recall by two independent evaluators who were blinded to the study. Using the two-stage precision validation, we evaluated all entries for correctness and operational status. During stage one, automated information retrieval identified 49,637 entities associated with the 34,475 candidate FP locations, followed by manual verification (stage two). Our results demonstrated a precision of 91.6%, where 31,593 out of 34,475 were valid. The remaining 2,882 entries were determined to be invalid, either because the organization no longer existed, had closed or discontinued food assistance services, or was misclassified as a FP. To further ensure accuracy, we assessed inter-rater agreement in a random subsample, which demonstrated 93% agreement betweenevaluators, the remaining 7% of discrepancies reflected differences in organizational naming at the same verified FP address rather than disagreement about FP status.

For recall, each evaluator created a gold standard dataset of 150 FPs. For the first evaluator, 121 matches were identified within our FP dataset, yielding a recall of 0.807. For the second evaluator, 114 matches were identified within our FP dataset, yielding a recall of 0.760. After combining both evaluators’ datasets and eliminating duplicates, the recall was 0.758, corresponding to 179 matches out of 236 unique entities.

We conducted an error analysis of the unmatched entities, as detailed in Supplementary Fig. 1. From the first evaluator’s dataset, 23 were food banks, which typically distribute to FPs rather than to individuals, two provided drive-thru services, one was a mobile pantry, and the remaining three were typical FPs that were missed by our dataset. From the second evaluator’s dataset, 16 were food banks, seven had religious affiliations, one catered primarily to college students, one initiated operations after we built the FP dataset, one had recently changed its location, and the remaining 10 were typical FPs that were missed by our dataset. Excluding food banks, the recall improved to 0.952 and 0.851 for the two gold standards.

### Accessibility analysis: urban and rural perspectives

Table 1a and b detail the FP accessibility statistics and characteristics for the 198,767 BGs in urban areas and 41,013 BGs in rural areas, respectively. For each BG, we categorized FP accessibility as high, medium, or low access based on walking/public transit time to the nearest FP in urban areas and driving distance to the nearest FP in rural areas. Detailed accessibility criteria can be found in the Methods section.

**Table 1:** Summary statistics of FP accessibility and characteristics of BGs in (a) urban and (b) rural areas in the U.S.

Variable	Overall	Low access^a,b^	Medium access^a,b^	High access^a,b^
(a)				
Number of BGs in Urban Areas [number (proportion of total)]	198,767	52,714 (26.5)	48,414 (24.4)	97,639 (49.1)
ADI [mean (95%CI)]	45.8 (45.6, 45.9)	44.3 (44.1, 44.5)	42.6 (42.3, 42.8)	48.1 (47.9, 48.3)
Travel time (in minutes) by walking/public transit [mean (95%CI)]	60.1 (58.9, 61.3)	159.6 (155.1, 164.0)	42.2 (42.1, 42.3)	16.1 (16.0, 16.1)

(b)				
Number of BGs in Rural Areas [number (proportion of total)]	41,013	3,502 (8.5)	8,029 (19.6)	29,482 (71.9)
ADI [mean (95%CI)]	71.3 (71.1, 71.5)	73.2 (72.4, 73.9)	72.6 (72.2, 73.1)	70.7 (70.4, 70.9)
Travel distance (in miles) [mean (95%CI)]	8.0 (7.8, 8.3)	41.2 (37.1, 45.2)	13.9 (13.8, 13.9)	3.5 (3.4, 3.5)

^a^Access categories for BGs in urban area were defined as low access (No walking routes within 30 min and no public transit within 60 min), medium access (Nearest FP between 15–30 min by walking or 30–60 min by public transit) and high access (Nearest FP within 15 min by walking or 30 min by public transit).

^b^Access categories for BGs in rural area were defined as low access (No FP within 20-mile driving distance), medium access (Nearest FP between 10–20-mile driving distance) and high access (Nearest FP within 10-mile driving distance).

Table 1a shows that in urban areas 97,639 (49.1%), 48,414 (24.4%), and 52,714 (26.5%) of BGs have high, medium, and low access, respectively, to their nearest FPs. Table 1b shows that in rural areas 29,482 (71.9%), 8,029 (19.6%), and 3,502 (8.5%) of BGs have high, medium, and low access, respectively, to their nearest FPs. On average, BGs in rural areas are more socioeconomically disadvantaged than BGs in urban areas (ADI 71.3 vs. 45.8, respectively).

**Fig. 1:**
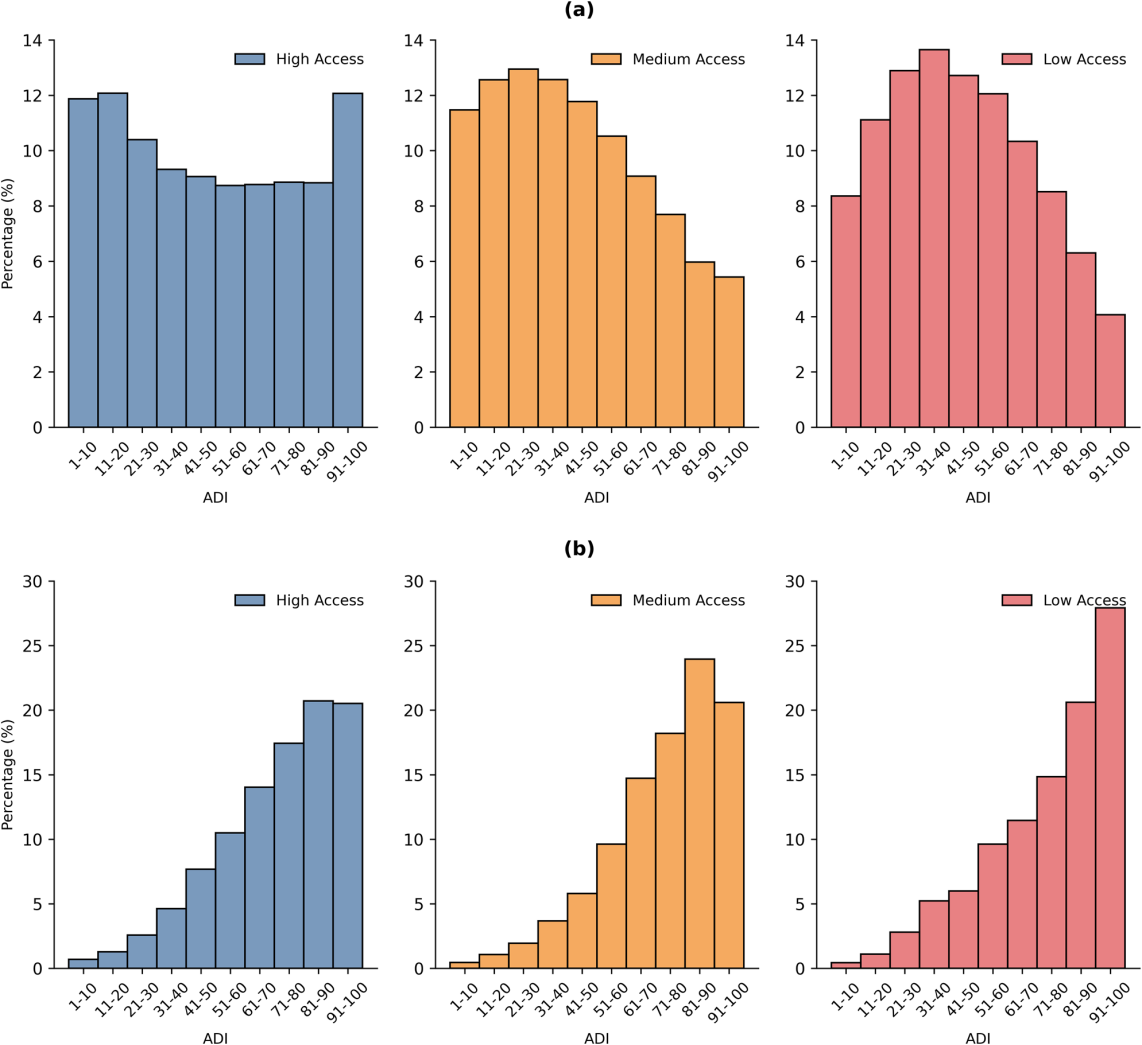
The distribution of BGs by ADI, organized by FP accessibility category in (a) urban and (b) rural areas. Note that the BG percentages within the same FP accessibility category (i.e., same-colored bars) sum to 100%.

Figure 1a shows the distribution of BGs by ADI in urban areas, organized by FP accessibility category. For BGs with medium and low access to FPs, the distribution is skewed toward lower ADIs. For BGs with high access to FPs, there is an approximately U-shaped distribution. Figure 1b shows the corresponding distributions for BGs in rural areas. The distribution of BGs is heavily skewed toward higher ADIs across all three FP access levels, indicating significant socioeconomic vulnerability. In rural areas, over 25% of BGs with low access to FPs have an ADI between 90 and 100 whereas only 4% of BGs with low FP access fall within this ADI range in urban areas.

**Fig. 2:**
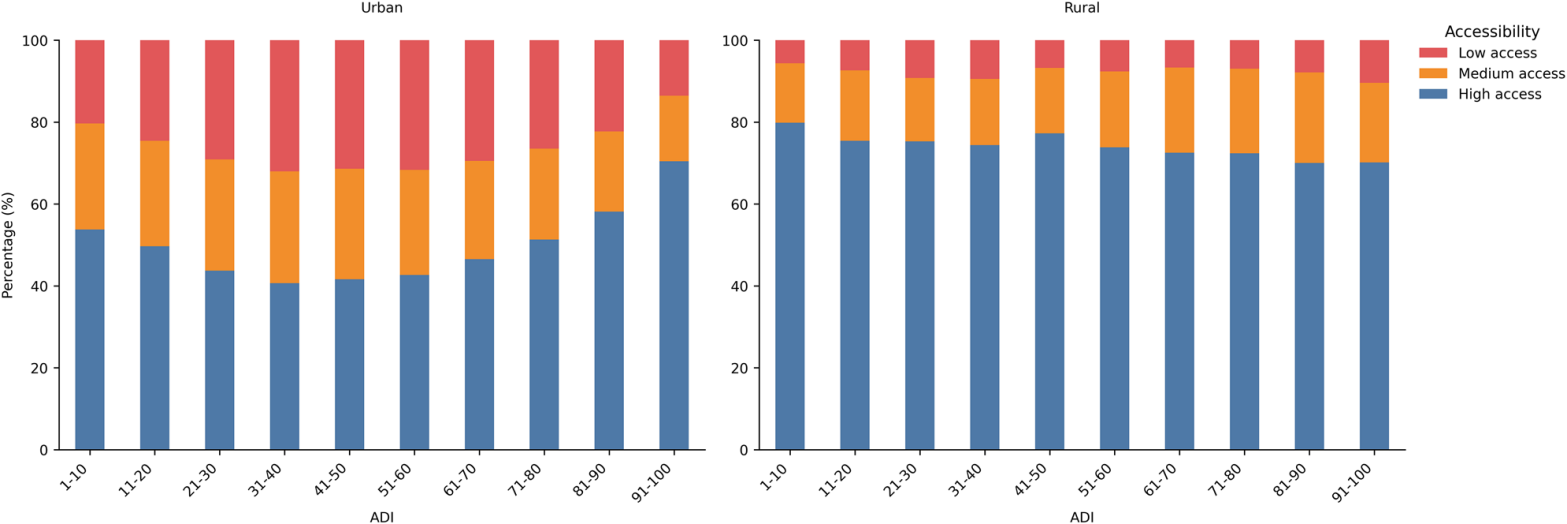
The distribution of BGs by FP accessibility category, organized by ADI in urban and rural areas.

Figure 2 shows the distribution of BGs by FP accessibility category, organized by ADI in urban and rural areas. In both urban and rural areas, high-access BGs constitute the largest proportion within each ADI range, but they have different trends. In urban areas, the proportion of BGs with high access to FPs is U-shaped. The opposite pattern is seen for BGs with low access to FPs. This indicates that the highest occurrence of BGs with low access to FPs is in areas of lower socioeconomic vulnerability. The proportion of BGs with medium access to FPs gradually decreases at the higher ADI ranges. In rural areas, the proportion of BGs with high access to FPs remains relatively stable across all ADI ranges, with a subtle decreasing trend from lower to higher ADI. Low-access BGs make up a small proportion overall but show a slight increase in both the mid-range (31–70) and the highest ADI range(91–100), suggesting that even in rural settings, areas with higher deprivation may face greater challenges in accessing FPs.

Table 2a, b present state-level FP accessibility in urban and rural areas, respectively. In urban areas (Table 2a), the five states with the highest FP access were New York (69.1% of BGs with high access), Hawaii, Rhode Island, Oregon, and Illinois. States with the lowest access were concentrated in the South, including West Virginia (52.3% of BGs with low access), Mississippi, South Carolina, Alabama, and Louisiana. Across states with low FP access, mean ADI values for low-access BGs consistently exceeded those in high-access states, indicating that urban areas with limited FP access tend to have greater socioeconomic vulnerability.

**Table 2:** Summary statistics of top-five states with high and low access to FPs in (a) urban and (b) rural areas.

(a)					
States with the Highest FP Accessibility in Urban Areas					
State	No. High-Access BGs	No. BGs	Percentage of High-Access BGs	Mean ADI of High-Access BGs	Mean ADI of all BGs
New York	10,149	14,688	69.1%	26.9	30.0
Hawaii	609	883	69.0%	13.2	11.7
Rhode Island	539	792	68.1%	46.1	41.1
Oregon	1493	2,338	63.9%	23.3	31.1
Illinois	5,158	8,362	61.7%	54.1	51.3
States with the Lowest FP Accessibility in Urban Areas					
State	No. Low-Access BGs	No. BGs	Percentage of Low-Access BGs	Mean ADI of Low-Access BGs	Mean ADI of all BGs
West Virginia	516	986	52.3%	69.0	71.5
Mississippi	528	1,050	50.3%	63.4	69.5
South Carolina	1,355	2,746	49.3%	59.9	62.2
Alabama	1,333	2,901	45.9%	62.4	67.9
Louisiana	1,581	3,510	45.0%	64.5	62.5

(b)					
States with the Highest FP Accessibility in Rural Areas					
State	No. High-Access BGs	No. BGs	Percentage of High-Access BGs	Mean ADI of High-Access BGs	Mean ADI of all BGs
Connecticut	177	180	98.3%	39.7	39.4
Massachusetts	91	95	95.8%	34.3	34.0
New Hampshire	388	427	90.9%	49.6	49.3
Vermont	331	387	85.5%	53.9	53.3
Indiana	1,126	1,329	84.7%	74.8	74.7
States with the Lowest FP Accessibility in Rural Areas					
State	No. Low-Access BGs	No. BGs	Percentage of Low-Access BGs	Mean ADI of Low-Access BGs	Mean ADI of all BGs
Alaska	83	180	46.1%	66.1	49.1
Arizona	102	295	34.6%	88.7	76.4
Nevada	73	216	33.8%	66.0	53.1
South Dakota	121	390	31.0%	80.5	70.7
California	213	741	28.7%	42.5	39.1

**Fig. 3:**
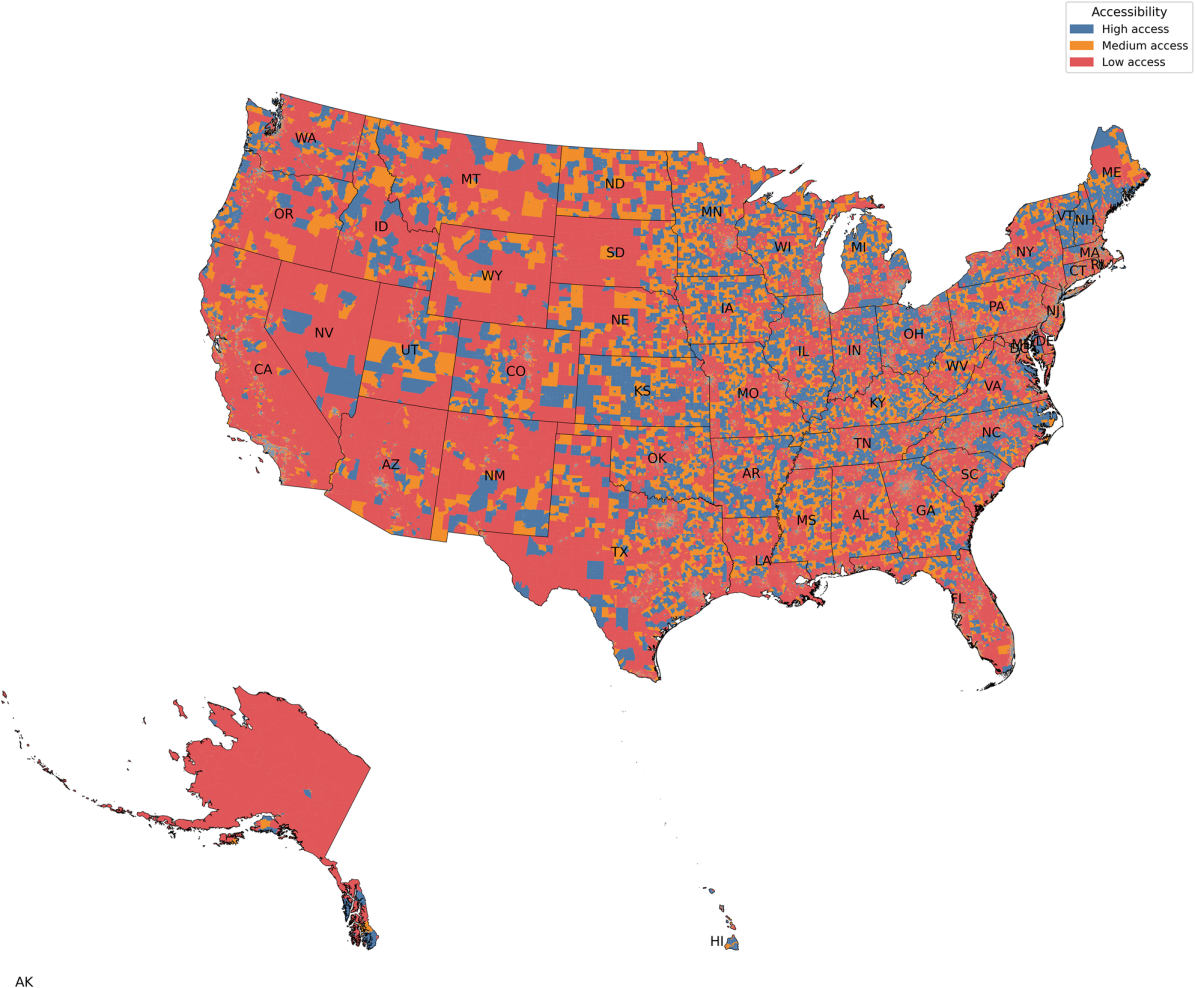
Nationwide accessibility to FPs at the BG level. (Map generated by Python 3.12).

In rural areas (Table 2b), four of the five highest-access states were in the Northeast (Connecticut, Massachusetts, New Hampshire, and Vermont), with Connecticut leading at 98.3% of BGs with high access. States with the lowest access were concentrated in the West (Alaska, Arizona, Nevada, and California). Alaska had the highest proportion of low-access BGs (46.1%), with a mean ADI (66.1) substantially higher than the state mean (49.1). Mean ADI values for low-access BGs exceeded state averages across all low-access states, indicating an association between limited FP access and higher socioeconomic vulnerability. For detailed state-level data, see Fig. 3 and Supplementary Figs. 2–7.

Table 3a, b summarize demographic characteristics by FP accessibility in urban and rural areas, respectively. In urban areas (Table 3a), high-access BGs had younger populations (22.8% aged 18– < 30 vs. 16.5% in low-access areas), greater racial/ethnic diversity, including lower proportions of non-Hispanic White residents (48.6% vs. 72.9%), and higher proportions of non-Hispanic Black (17.9% vs. 7.6%) and Hispanic/Latino (22.6% vs. 11.8%) residents. This pattern suggests FP placement may align with communities experiencing greater food insecurity risk, given that Hispanic and Black populations are disproportionately affected in the U.S. Rural areas showed different patterns (Table 3b). While high-access BGs were also younger (18.8% aged 18– < 30 vs. 16.3% in low-access areas), low-access BGs in rural areas were more racially diverse, with higher proportions of Hispanic/Latino (11.3% vs. 8.3% overall) and non-Hispanic Native American residents (7.2% vs. 1.8% overall). These findings indicate that in rural areas, limited FP access is more commonly experienced in older and more racially diverse communities.

**Table 3:** Summary statistics of FP accessibility and demographic characteristics of BGs in (a) urban and (b) rural areas in the U.S.

Variable	Overall	Low access^a,b^	Medium access^a,b^	High access^a,b^
(a)				
Percent (%) of residents in each census block groups [mean (95% CI)]				
Age				
18– < 30	20.3 (20.3, 20.4)	16.5 (16.4, 16.6)	19.4 (19.3, 19.5)	22.8 (22.7, 22.9)
30– < 40	17.7 (17.6, 17.7)	15.6 (15.6, 15.7)	17.2 (17.1, 17.3)	18.9 (18.9, 19.0)
40– < 50	16.1 (16.1, 16.1)	16.7 (16.7, 16.8)	16.3 (16.2, 16.3)	15.7 (15.6, 15.7)
50– < 60	16.9 (16.9, 17.0)	18.6 (18.6, 18.7)	17.1 (17.0, 17.2)	15.9 (15.9, 16.0)
60– < 65	8.3 (8.2, 8.3)	9.2 (9.2, 9.3)	8.3 (8.3, 8.4)	7.7 (7.7, 7.8)
65 and older	20.8 (20.7, 20.8)	23.3 (23.2, 23.4)	21.8 (21.6, 21.9)	18.9 (18.9, 19.0)
Gender				
Male	48.6 (48.6, 48.7)	49.4 (49.4, 49.5)	48.4 (48.3, 48.4)	48.3 (48.3, 48.4)
Female	51.4 (51.3, 51.4)	50.6 (50.5, 50.6)	51.6 (51.6, 51.7)	51.7 (51.6, 51.7)
Race/Ethnicity				
Non-Hispanic White	57.9 (57.7, 58.0)	72.9 (72.6, 73.1)	60.4 (60.1, 60.6)	48.6 (48.4, 48.8)
Non-Hispanic Black	13.5 (13.4, 13.6)	7.6 (7.5, 7.7)	11.2 (11.1, 11.4)	17.9 (17.7, 18.0)
Non-Hispanic Native American	0.4 (0.4, 0.4)	0.5 (0.5, 0.6)	0.3 (0.3, 0.4)	0.4 (0.4, 0.4)
Non-Hispanic Asian	5.9 (5.8, 5.9)	4.1 (4.0, 4.1)	6.6 (6.5, 6.7)	6.5 (6.4, 6.6)
Non-Hispanic Other	3.8 (3.7, 3.8)	3.2 (3.2, 3.3)	3.8 (3.7, 3.8)	4.1 (4.0, 4.1)
Hispanic/Latino	18.6 (18.4, 18.7)	11.8 (11.6, 12.0)	17.7 (17.4, 17.9)	22.6 (22.4, 22.8)

(b)				
Percent (%) of residents in each census block groups [mean (95% CI)]				
Age				
18– < 30	18.0 (17.9, 18.2)	16.3 (16.0, 16.6)	15.9 (15.7, 16.1)	18.8 (18.7, 19.0)
30– < 40	14.8 (14.7, 14.9)	14.1 (13.8, 14.4)	13.9 (13.7, 14.1)	15.1 (15.0, 15.2)
40– < 50	14.6 (14.6, 14.7)	14.1 (13.8, 14.3)	14.6 (14.4, 14.8)	14.7 (14.6, 14.8)
50– < 60	17.3 (17.2, 17.4)	17.4 (17.1, 17.6)	18.2 (18.1, 18.4)	17.0 (16.9, 17.1)
60– < 65	9.6 (9.5, 9.6)	10.1 (9.9, 10.2)	10.2 (10.1, 10.4)	9.3 (9.3, 9.4)
65 and older	25.7 (25.6, 25.8)	28.1 (27.7, 28.5)	27.2 (26.9, 27.4)	25.0 (24.9, 25.2)
Gender				
Male	49.6 (49.5, 49.6)	50.6 (50.3, 50.9)	50.3 (50.2, 50.5)	49.2 (49.2, 49.3)
Female	50.4 (50.4, 50.5)	49.4 (49.1, 49.7)	49.7 (49.5, 49.8)	50.8 (50.7, 50.9)
Race/Ethnicity				
Non-Hispanic White	78.0 (77.8, 78.3)	70.1 (69.0, 71.1)	82.7 (82.2, 83.2)	77.7 (77.4, 78.0)
Non-Hispanic Black	8.0 (7.9, 8.2)	8.0 (7.4, 8.7)	7.1 (6.7, 7.4)	8.3 (8.1, 8.5)
Non-Hispanic Native American	1.8 (1.7, 1.9)	7.2 (6.4, 7.9)	1.5 (1.3, 1.7)	1.3 (1.2, 1.3)
Non-Hispanic Asian	0.9 (0.8, 0.9)	0.7 (0.6, 0.8)	0.5 (0.4, 0.5)	1.0 (1.0, 1.0)
Non-Hispanic Other	3.0 (2.9, 3.0)	2.7 (2.6, 2.9)	2.6 (2.5, 2.7)	3.1 (3.1, 3.2)
Hispanic/Latino	8.3 (8.1, 8.4)	11.3 (10.7, 12.0)	5.7 (5.4, 6.0)	8.7 (8.5, 8.8)

^a^Access categories for BGs in urban area were defined as low access (No walking routes within 30 min and no public transit within 60 min), medium access (Nearest FP between 15–30 min by walking or 30–60 min by public transit) and high access (Nearest FP within 15 min by walking or 30 min by public transit).

^b^Access categories for BGs in rural area were defined as low access (No FP within 20-mile driving distance), medium access (Nearest FP between 10–20-mile driving distance) and high access (Nearest FP within 10-mile driving distance).

Tables 4a, b and 5a, b present education, employment, household composition, income, poverty status, and public assistance data stratified by FP accessibility. In urban areas (Tables 4a and 5a), high-access BGs demonstrated indicators of greater socioeconomic vulnerability: lower educational attainment (13.8% without high school diploma vs. 8.6% in low-access areas), higher unemployment (4.2% vs. 2.8% in low-access areas), more single-parent households (13.3% single female householders with children vs. 6.6% in low-access areas), lower incomes (41.9% earning < $50,000 vs. 29.6% in low-access areas), higher poverty rates (16.0% vs. 8.6%), and greater public assistance participation (18.6% vs. 8.6%). This pattern indicates that FPs in urban areas are more concentrated in socioeconomically vulnerable communities.

**Table 4:** Summary statistics of education attainment, labor force status, and household composition of BGs stratified by FP accessibility in (a) urban and (b) rural areas in the U.S.

Variable	Overall	Low access^a,b^	Medium access^a,b^	High access^a,b^
(a)				
Percent (%) of residents in each census block groups [mean (95% CI)]				
Education attainment				
Less than high school	11.3 (11.3, 11.4)	8.6 (8.5, 8.7)	9.4 (9.3, 9.5)	13.8 (13.7, 13.9)
High school or some college	54.0 (53.9, 54.1)	56.2 (56.1, 56.4)	53.0 (52.8, 53.1)	53.3 (53.2, 53.4)
Bachelor’s degree or higher	34.7(34.6, 34.8)	35.2 (35.0, 35.3)	37.6 (37.4, 37.8)	33.0 (32.8, 33.1)
Labor force				
Employed	60.2 (60.1, 60.3)	59.0 (58.9, 59.1)	60.9 (60.8, 61.0)	60.5 (60.4, 60.6)
Unemployed	3.6 (3.6, 3.6)	2.8 (2.8, 2.9)	3.3 (3.3, 3.3)	4.2 (4.2, 4.2)
Other	0.4 (0.4, 0.4)	0.6 (0.5, 0.6)	0.5 (0.5, 0.5)	0.3 (0.3, 0.3)
Not in labor force	35.8 (35.7, 35.9)	37.6 (37.4, 37.7)	35.3 (35.2, 35.5)	35.1 (35.0, 35.2)
Household Composition				
Married couple with children	27.1 (27.0, 27.1)	29.8 (29.6, 29.9)	28.8 (28.6, 28.9)	24.8 (24.6, 24.9)
Married couple without children	44.0 (43.9, 44.1)	50.7 (50.5, 50.8)	45.4 (45.2, 45.6)	39.7 (39.6, 39.8)
Single female householder with children	10.6 (10.5, 10.6)	6.6 (6.5, 6.7)	9.3 (9.2, 9.4)	13.3 (13.2, 13.4)
Single female householder without children	10.3 (10.3, 10.4)	6.9 (6.8, 7.0)	9.2 (9.1, 9.3)	12.7 (12.6, 12.8)
Single male householder with children	3.5 (3.5, 3.6)	2.9 (2.8, 2.9)	3.3 (3.2, 3.4)	4.0 (4.0, 4.1)
Single male householder without children	4.6 (4.5, 4.6)	3.2 (3.2, 3.3)	4.0 (4.0, 4.1)	5.5 (5.5, 5.6)

(b)				
Percent (%) of residents in each census block groups [mean (95% CI)]				
Education attainment				
Less than high school	12.6 (12.5, 12.7)	14.3 (13.9, 14.6)	13.1 (12.9, 13.3)	12.3 (12.2, 12.4)
High school or some college	66.7 (66.6, 66.8)	67.5 (67.1, 67.9)	68.5 (68.2, 68.7)	66.1 (66.0, 66.3)
Bachelor’s degree or higher	20.7 (20.5, 20.8)	18.3 (17.9, 18.7)	18.4 (18.2, 18.7)	21.6 (21.4, 21.7)
Labor force				
Employed	53.7 (53.6, 53.8)	49.5 (49.0, 50.0)	52.3 (52.0, 52.5)	54.6 (54.4, 54.7)
Unemployed	3.1 (3.0, 3.1)	3.3 (3.1, 3.4)	2.9 (2.8, 3.0)	3.1 (3.1, 3.2)
Other	0.2 (0.2, 0.3)	0.1 (0.1, 0.2)	0.2 (0.1, 0.2)	0.3 (0.2, 0.3)
Not in labor force	43.0 (42.9, 43.1)	47.1 (46.6, 47.6)	44.7 (44.4, 45.0)	42.1 (41.9, 42.2)
Household Composition				
Married couple with children	24.0 (23.9, 24.2)	22.2 (21.8, 22.7)	24.0 (23.7, 24.2)	24.3 (24.1, 24.4)
Married couple without children	50.5 (50.3, 50.6)	53.3 (52.7, 54.0)	54.5 (54.2, 54.9)	49.0 (48.8, 49.3)
Single female householder with children	9.4 (9.3, 9.5)	8.2 (7.9, 8.6)	7.0 (6.8, 7.2)	10.2 (10.0, 10.3)
Single female householder without children	8.5 (8.4, 8.6)	8.7 (8.4, 9.1)	7.6 (7.4, 7.8)	8.7 (8.6, 8.8)
Single male householder with children	3.8 (3.8, 3.9)	3.5 (3.3, 3.7)	3.2 (3.1, 3.3)	4.0 (3.9, 4.1)
Single male householder without children	3.8 (3.8, 3.9)	4.0 (3.8, 4.3)	3.7 (3.5, 3.8)	3.8 (3.8, 3.9)

^a^Access categories for BGs in urban area were defined as low access (No walking routes within 30 min and no public transit within 60 min), medium access (Nearest FP between 15–30 min by walking or 30–60 min by public transit) and high access (Nearest FP within 15 min by walking or 30 min by public transit).

^b^Access categories for BGs in rural area were defined as low access (No FP within 20-mile driving distance), medium access (Nearest FP between 10–20-mile driving distance) and high access (Nearest FP within 10-mile driving distance).

**Table 5:** Summary statistics of household income, poverty status, and public assistance stratified by food pantry (FP) accessibility in (a) urban and (b) rural areas in the U.S.

Variable	Overall	Low access^a,b^	Medium access^a,b^	High access^a,b^
(a)				
Percent (%) of residents in each census block groups [mean (95% CI)]				
Yearly Household Income				
Less than $50,000	36.2 (36.1, 36.3)	29.6 (29.4, 29.7)	31.9 (31.8, 32.1)	41.9 (41.7, 42.0)
$50,000 or more	63.8 (63.7, 63.9)	70.4 (70.3, 70.6)	68.1 (67.9, 68.3)	58.2 (58.0, 58.3)
Household poverty status in the past 12 months				
Below poverty level	12.6 (12.6, 12.7)	8.6 (8.6, 8.7)	10.1 (10.0, 10.2)	16.0 (15.9, 16.1)
At or above poverty level	87.4 (87.3, 87.4)	91.4 (91.3, 91.4)	89.9 (89.8, 90.0)	84.0 (83.9, 84.1)
Cash public assistance income or households receiving Food Stamps/SNAP benefits in the past 12 months				
With cash public assistance or Food Stamps/SNAP	14.1 (14.0, 14.2)	8.6 (8.5, 8.7)	11.1 (10.9, 11.2)	18.6 (18.4, 18.7)
Without cash public assistance or Food Stamps/SNAP	85.9 (85.8, 86.0)	91.4 (91.3, 91.5)	88.9 (88.8, 89.1)	81.4 (81.3, 81.6)

(b)				
Percent (%) of residents in each census block groups [mean (95% CI)]				
Yearly Household Income				
Less than $50,000	47.7 (47.5, 47.9)	50.2 (49.6, 50.8)	46.7 (46.4, 47.1)	47.7 (47.8, 47.9)
$50,000 or more	52.3 (52.1, 52.5)	49.8 (49.2, 50.4)	53.3 (52.9, 53.6)	52.3 (52.1, 52.5)
Household poverty status in the past 12 months				
Below poverty level	15.7 (15.6, 15.8)	17.3 (16.9, 17.8)	14.7 (14.5, 15.0)	15.8 (15.7, 15.9)
At or above poverty level	84.3 (84.2, 84.4)	82.7 (82.2, 83.1)	85.3 (85.0, 85.5)	84.2(84.1, 84.4)
Cash public assistance income or households receiving Food Stamps/SNAP benefits in the past 12 months				
With cash public assistance or Food Stamps/SNAP	14.7 (14.6, 14.9)	14.9 (14.4, 15.4)	12.9 (12.6, 13.1)	15.2 (15.0, 15.4)
Without cash public assistance or Food Stamps/SNAP	85.3 (85.2, 85.4)	85.1 (84.6, 85.6)	87.1 (86.9, 87.4)	84.8 (84.6, 85.0)

^a^Access categories for BGs in urban area were defined as low access (No walking routes within 30 min and no public transit within 60 min), medium access (Nearest FP between 15–30 min by walking or 30–60 min by public transit) and high access (Nearest FP within 15 min by walking or 30 min by public transit).

^b^Access categories for BGs in rural area were defined as low access (No FP within 20-mile driving distance), medium access (Nearest FP between 10–20-mile driving distance) and high access (Nearest FP within 10-mile driving distance).

Rural areas (Tables 4b and 5b) showed more complex patterns. While high-access BGs in rural areas had higher educational attainment (21.6% with bachelor’s degree or higher vs. 18.3% in low-access areas) and employment rates (54.6% vs. 49.5%), differences in household income, poverty rates, and public assistance participation were modest across access levels. For instance, poverty rates ranged from 14.7% in medium-access areas to 17.3% in low-access areas, compared to 15.7% overall. These findings suggest that in rural areas, FP placement is not as tightly linked to socioeconomic indicators as in urban areas, and limited access may reflect geographic barriers rather than strategic placement based on community need.

### Statistical approaches

We used generalized linear regression models to examine associations between BG characteristics and access to FPs, measured by log-transformed travel time (urban) or driving distance (rural). Full regression results are provided in Supplementary Tables 1 to 10.

Across both urban and rural areas, several consistent patterns were observed. FPs were more accessible in BGs with greater socioeconomic vulnerability, as indicated by higher ADI, younger age composition (18– < 40 years), higher proportions of non-Hispanic Black and Hispanic/Latino residents, lower household incomes, higher poverty rates, and greater public assistance participation. These associations persisted after adjustment for land area, suggesting that FP placement is broadly aligned with community need.

Important differences were observed between urban and rural areas in educational attainment, employment, and minority populations. In urban areas, FPs were more accessible in BGs with lower educational attainment, higherunemployment, and higher proportions of single-parent households, especially those headed by single females. These patterns are consistent with targeted placement in high-need communities. In rural areas, these relationships were weaker or in the opposite direction. Higher educational attainment and greater employment were associated with shorter travel distances, indicating that rural FP placement may be influenced more by geographic and infrastructure constraints than by need-based targeting. In both urban and rural areas, BGs with higher proportions of individuals not in the labor force had poorer FP access. Associations with Native American populations were small, with slightly longer travel times in urban areas and no significant differences in rural areas.

Gender composition also showed consistent patterns. BGs with higher proportions of female residents had better FP access, whereas higher male composition was associated with longer travel times or distances. Although effect sizes were generally small, likely reflecting the large national sample, the overall findings suggest stronger alignment between FP placement and socioeconomic vulnerability in urban areas than in rural areas.

## Discussion

### Geographic disparity

We identified variation in FP accessibility both across states and within states. While prior research has documented variation in FP density across states (2.60 to 7.76 FPs per 100,000 people)^39^, our study is among the first to assess BG-level accessibility at a national scale. State-level accessibility provides a national overview of FP accessibility. Our results show that Northeastern states generally demonstrate higher FP accessibility than states in the South and West. This geographic pattern may compound existing regional disparities in food insecurity, as the South experiences both the highest food insecurity rate (14.7%) and relatively lower FP accessibility^22^. Within-state accessibility is also important. A state may appear to have adequate FP density yet still experience poor accessibility if facilities are clustered in certain locations, leaving other communities underserved. For example, although prior research reported that Alabama has relatively high FP density, with 8.68 FPs per 100,000 people in urban areas and 6.11 FPs per 100,000 people in rural areas^39^, our findings for Alabama show that 45.9% of BGs in urban areas and 11.7% of BGs in rural areas have low FP access. In addition, the level of socioeconomic disadvantage among low-access BGs differs substantially: low-access rural BGs in Alabama have an average ADI of 86.5, indicating severe socioeconomic disadvantage, compared with a mean ADI of 62.4 among low-access urban BGs. These results highlight that states should consider not only where low access occurs but also prioritize most socioeconomically disadvantaged communities. Given that food insecurity is more prevalent in the South and FP access also tends to be lower in that region, targeted strategies are needed to improve FP coverage and reduce food insecurity in these high-need areas.

### Urban and rural differences

Our finding that rural areas have proportionally higher FP accessibility than urban areas (71.9% versus 49.1% of BGs with high access) is consistent with prior work showing that the number of charitable food locations per 1,000 people is highest in counties classified as completely rural according to urban–rural continuum codes^40^. One study conducted in Minnesota similarly found no association between living in a small town or rural area and self-reported barriers to pantry use^41^. In contrast, a regional study from Indiana reported higher frequencies of transportation- and stigma-related barriers in rural compared with urban areas^42^. Together, these findings suggest that accessibility alone does not fully capture barriers to FP utilization. Although a majority of BGs in rural areas in our study are classified as having high accessibility, the 28.1% with medium or low access represent substantial populations facing geographic constraints, and even BGs with high accessibility may encounter practical obstacles, such as limited FP operating hours, that restrict regular use.

### Accessibility and demographic and socioeconomic variables

Our findings reveal distinct patterns in the alignment between FP accessibility and BG-level sociodemographic characteristics across urban and rural areas. In urban areas, FP placement showed strong associations with multiple indicators of need including lower income, higher poverty rates, greater SNAP participation, and higher proportions of racial/ethnic minority residents and single-parent households. These patterns suggest that FP placement in urban settings may be more responsive to community needs, potentially reflecting strategic efforts to locate services in areas with higher levels of food insecurity risk. This is consistent with prior findings showing that FPs are more likely to locate in areas with high levels of poverty and high-minority neighborhoods in urban areas^20,21^.

In rural areas, while FPs were also more accessible in areas with higher ADI overall, the relationships with specific socioeconomic indicators were weaker and sometimes reversed. Notably, rural communities with lower educational attainment and higher proportions of residents not in the labor force actually had longer travel distances to FPs. This suggests that rural FP placement may be shaped more by infrastructure constraints, population density, and volunteer availability than by strategic targeting of the most vulnerable populations. Overall, the findings highlight a stronger alignment between FP accessibility and social vulnerability in urban areas, while rural communities, particularly those with aging populations and limited public assistance participation, may remain underserved despite elevated need.

### Strengths and limitations

Our study has several strengths. First, we created a national FP dataset paired with systematic two-stage validation, enabling the first nationwide characterization of accessibility at the BG level. Second, we conducted a comprehensive, nationwide analysis of food access disparities. This provides a broad picture of FP accessibility issues, allowing policymakers and researchers to approach each state’s challenges with greater specificity and context. Third, we made comparative analyses of both urban and rural areas, highlighting specific patterns that might be overlooked in broader studies that do not distinguish between these two settings. We also examined ADI and detailed demographic and socioeconomic characteristics in combination with FP access, providing deeper insights into the complex relationship between social vulnerability and FP accessibility. Finally, our work establishes a foundation for future research in this field, especially for studies exploring longitudinal changes in food access and evaluating the impact of future policies.

Our study has several limitations. First, although we utilized a large-scale dataset, prior research has shown that secondary food environment data sources are often inaccurate, incomplete, out of date, or contain geospatial errors^43–46^. While prior regional studies have the capacity to supplement downloaded data with several local sources and conduct direct phone calls or on-site visits to verify operating status^16,47^, the national scope of our dataset (n = 34,475) precluded direct contact or physical ground-truthing for the entire sample. Instead, we conducted a systematic two-stage validation of all FP listings using publicly available online sources. As a result, some residual misclassification may remain, particularly for organizations with limited or outdated online information. This limitation is consistent with prior research showing that food pantry directories often contain inaccuracies related to operating status when relying on online sources^16,48^. Second, our dataset relies on publicly available listings and online presence, which may not capture the full universe of FPs. Prior studies have similarly noted that smaller, informal, or newly established food assistance sites are often underrepresented in secondary datasets^21,49^. These challenges may be amplified during periods of rapid change, such as the COVID-19 pandemic, when many FPs opened or closed in response to shifting demand. Although our two-stage validation improved data accuracy, these structural limitations of online-based FP datasets cannot be fully eliminated. Third, although FPs in the U.S. generally provide food at no cost, we were unable to verify this operational detail for every location due to the lack of standardized, publicly available information. The dataset also lacks key operational characteristics,including food availability, nutritional quality of offerings, and does not consistently distinguish between food pantries, food banks, soup kitchens, or hybrid service models. Our evaluation indicates that a small proportion of food banks (less than 1.5%) remain in the dataset, reflecting persistent classification challenges inherent in heterogeneous public data sources. Finally, the dataset is cross-sectional and reflects food pantry accessibility in 2022, limiting our ability to assess temporal changes in FP availability or access. Because our analysis targeted large-scale accessibility of FPs across the U.S., these limitations prevent us from reproducing previously validated spatiotemporal accessibility to FPs that were conducted in small regions^50^.

### Implications for policy and practice

Our findings have several important implications. First, state-level variation in FP accessibility indicates that solutions must be tailored to local contexts rather than implemented uniformly. States with clear accessibility gaps, such as West Virginia and Alaska, may require targeted investment in FP development through approaches such as public–private partnerships, mobile pantry programs, or integration with existing rural service delivery networks. Allocation of federal resources through programs such as TEFAP, as well as foundation funding priorities, should consider both food insecurity prevalence and accessibility when targeting support. Future research should examine why some high-need states have developed more accessible FP networks than others. Comparative case studies of state policies, nonprofit infrastructure, and community organizing capacity may help identify transferable strategies for improving access in underserved regions.

Second, the observed urban–rural differences in socioeconomic status suggest that rural communities may require additional support to ensure that FPs reach the most vulnerable neighborhoods. Economic instability in rural regions, including job loss, population decline, and aging demographics, exacerbates challenges related to limited food access^51^. Interventions may include GIS-based needs assessments to identify underserved areas, targeted funding to support FP development in high-need rural communities, and transportation assistance programs to help clients access existing facilities.

Third, while our findings show that many FPs are located near vulnerable populations, geographic proximity alone does not ensure effective access. Prior research has shown that FPs often provide highly processed foods with limited nutritional value^48,52–54^, and that open pantries frequently have insufficient food supply to meet client demand^16^. In addition, FPs may not always operate reliably, prior work has found that only about 50% of pantries were open during the hours listed in online directories, with several having prolonged or indefinite closures^16^. Cultural barriers may also limit access, such as when English is not the primary language of household members while pantry staff exclusively speak English, or when the food offered is not culturally appropriate for certain households^55^. Stigma may prevent individuals from accessing pantry services, as they may be sensitive about how they might be perceived for visiting a pantry, have negative opinions of pantries as organizations, or hold poor perceptions of food quality^56,57^. Policymakers should consider these multidimensional barriers when designing interventions to improve food access.

Finally, we acknowledge the broader critique about the role of charitable food assistance in addressing food insecurity. Scholars have argued that FPs serve as stopgap measures that distract from addressing poverty and food insecurity as human rights issues^58^, and that reliance on emergency food systems allows governments to avoid addressing the root causes of hunger^59^. Our findings on geographic and socioeconomic disparities in FP access point to the limitations of charitable approaches and highlight the need for comprehensive policy solutions that address structural determinants of food insecurity.

## Methods

### FP dataset and validation

We created a national dataset of FPs using an information retrieval approach. First, we included FPs (n = 15,800) throughout the U.S. from the web-based, user-validated dataset “foodpantries.org”^60^. In addition, we collected potential FP locations from Google Maps. We searched each of the 3,143 counties in the U.S., using “food pantry” as the search query. As Google Maps often returned results unrelated to FPs, we used pantries or charity-related location types, such as “food pantries,” “food banks,” and “non-profit organizations,” as filters for the candidates. After removing duplicate addresses, these procedures identified 34,475 FPs.

We assessed the accuracy and comprehensiveness of the FP dataset through recall and precision. Precision was measured as the percentage of FPs in our dataset that are real FPs. Recall was measured as the percentage of FPs in the U.S. that are present in our dataset. For precision, we conducted a systematic two-stage verification process to determine whether each candidate listing represented an operational FP. Stage one involved automated information retrieval: for all 34,475 candidate locations, we used GPT-4o to perform an initial large-scale search using each site’s physical address. We retrieved publicly available online information associated with each address, including organizational name, website, service descriptions, and posted hours of operation. This automated step served only to collect candidate evidence, no inclusion or exclusion decisions were made at this stage. Stage two consisted of manual human verification. Research personnel reviewed the information from stage one to confirm the integrity of each entry. Two independent evaluators participated in this process. One evaluator conducted the full manual verification for all listings identified in stage one. To assess consistency and reliability, we then randomly selected 100 listings from this verified set and had a second evaluator independently evaluate them. A third reviewer subsequently assessed inter-rater agreement between the two evaluators and examined discrepancies. A listing was marked as invalid if it met any of the following criteria: (a) the website link was led to a non-existent organization; (b) the organization was confirmed to be permanently closed; or (c) the organization was determined to be an entity other than a FP.

To evaluate the recall of our FP dataset, the two evaluators independently identified FPs in the U.S. Evaluators were instructed to randomly select three FPs from each of the 50 states and District of Columbia using any method, such as internet searches on Google Maps and foodpantry.org, resulting in 150 FPs per evaluator. We calculated recall separately for each evaluator, and the overall recall based on the evaluators’ combined lists after removing duplicates.

### Urban and rural designation

The geographic data for BGs used in the study were based on the 2020 Census, which includes 239,780 BGs in the 50 states and the District of Columbia. We used the U.S. Census’s centers of population^61^ as the population-weighted centroid of each BG. To classify each BG into either rural or urban categories, we used the 2013 Rural–Urban Continuum Codes (RUCC)^62^ from the U.S. Department of Agriculture Economic Research Service. RUCC classifies urban and rural counties based on population size and adjacency to metropolitan areas. We used the first three (RUCC 1–3) metropolitan areas as urban and the remaining six (RUCC 4–9) non-metropolitan as rural to assign each BG a geographic type. We also incorporated 2020 BG geographic land area data from IPUMS National Historical Geographic Information System (NHGIS)^63^.

### Area deprivation index (ADI)

We incorporated 2020 ADI data from The Neighborhood Atlas^37,38^, which were updated in October 2022 using data from the 2016–2020 American Community Survey 5-year data summary. The ADI for each BG is calculated using 17 neighborhood-level measures, including income, education, employment, and housing quality. BGs are ranked based on their ADI both within each state (divided into deciles, scores range 1–10) and nationally (divided into percentiles, scores range 1–100), where higher scores indicate greater deprivation/disadvantage. In this study, the national percentile rankings were used as the ADI.

### Demographic and socioeconomicvariables

Demographic and socioeconomic variables at the BG level were obtained from the 2017–2021 American Community Survey (ACS) 5-year summary files, organized by the IPUMS NHGIS^64^. These variables include age group composition (percentages of residents aged 18– < 30, 30– < 40, 40– < 50, 50– < 60, 60– < 65, and 65 and older), gender composition (percent male and female), and racial/ethnic composition (percentages of non-Hispanic White, non-Hispanic Black, non-Hispanic American Indian/Alaska Native, non-Hispanic Asian, other non-Hispanic groups, and Hispanic/Latino residents). Educational attainment composition includes the percentage of individuals with less than a high school education, high school or some college, and a bachelor’s degree or higher. Labor force composition is defined by the proportion of individuals who are employed, unemployed, not in the labor force, or classified as "other." Yearly household income composition includes the percentage of households earning less than $50,000 versus $50,000 or more. Household composition includes six categories: married couple with children, married couple without children, single female householder with children, single female householder without children, single male householder with children, and single male householder without children. Additional indicators include household poverty status in the past 12 months (below vs. at or above the poverty level) and receipt of cash public assistance income or Food Stamps/SNAP benefits (with vs. without). Variables related to public assistance, household income, and poverty status are based specifically on 2021 ACS data.

### Accessibility criteria

In urban areas, FP accessibility was measured in relation to low-cost modes of intra-urban travel: walking and public transit. Public transit is typically a popular option for disadvantaged populations to reach destinations beyond walking distance^65^. FP accessibility was defined as the shorter of travel time by walking or public transit from the population-weighted centroid of each BG to the nearest FP. We categorized BGs as (1) high access: nearest FP within 15 min by walking or 30 min by public transit, (2) medium access: nearest FP within 15–30 min by walking or 30–60 min by public transit, or (3) low access: nearest FP more than 30 min by walking and more than 60 min by public transit, or no FP within 25 miles. These cutoff points are consistent with other food access studies^30,66,67^.

In rural areas, FP accessibility was measured in relation to driving distance, which aligns with the report by U.S. Department of Agriculture Economic Research Service, given that walking and public transport are typically not feasible options in rural areas, and the majority of U.S. households drive to access food resources in these areas^67^. FP accessibility was defined as the driving distance from the population-weighted centroid of each BG to the nearest FP. We categorized BGs as (1) high access: nearest FP within a 10-mile driving distance, (2) medium access: nearest FP within a 10–20 mile driving distance, or (3) low access: nearest FP more than 20 miles away or no FP within 25 miles. We also evaluated FP accessibility using travel time, categorizing BGs as (1) high access: nearest FP within a 15-min driving time, (2) medium access: nearest FP within a 15–30-min driving time, or (3) low access: nearest FP more than 30 min away or no FP within 25 miles; the results are presented in Supplementary Table 12.

For BGs in urban and rural areas, we computed the geodesic distance between the population-weighted centroid of each BG and the geographic coordinates of all FPs located within 25 miles. This process resulted in a list of 238,393 BGs for which their nearest FPs were identified. There are 1,387 BGs in total that do not have an FP within 25 miles; these were included in the low access category. Driving distances and travel times for walking and public transit were obtained using the Google Maps Distance Matrix API between the population-weighted centroid of each BG and its nearest FP. To ensure consistency, all travel times were calculated with the same start time on the same day. For public transit time, the calculation includes walking time to and from stops, waiting time, and the actual time spent on transit.

### Land area analysis

We analyzed the land area in our dataset for both urban and rural BGs (see Supplementary Table 11 and Supplementary Fig. 8). For urban BGs, the median land area was 0.99 km^2^, and 75% of BGs have an area below 3.34 km^2^. Similarly, rural BGs had larger land area (median 24.5 km^2^; and 75% below 88.8 km^2^), and their area distribution was also right skewed.

### Statistical approaches

*Outcome variables*: For linear regression analysis of BGs in urban areas, we created a measure of FP accessibility that represents the shortest travel time between each BG and its nearest FP by either walking or public transit. For BGs in rural areas, we created a measure of FP accessibility that represents the driving distance between each BG and its nearest FP. Since the travel time/distance data were right-skewed, distance to the nearest FP was log-transformed in all regression models to improve model fit.

*Covariates*: First, we designated ADI as a covariate for the first generalized linear regression model: *Model* 1: $${\widehat{Y}}_{travel time} ={\widehat{\beta }}_{intercept} +{\widehat{\beta }}_{ADI} {BG}_{ADI}$$ for the BGs in urban areas. Then, we built a single linear regression to estimate the impact of ADI percentiles of BGs on accessibility to FPs. $${\widehat{Y}}_{travel time}$$ is the log-transformed travel time to the nearest FP by walking or public transit, $${\beta }_{intercept}$$ is the coefficient estimate for the intercept of the regression model, and $${\beta }_{ADI}$$ is the mean estimates of the ADI of BGs ($${BG}_{ADI})$$.

For BGs in rural areas, we created the regression model: *Model* 2: $${\widehat{Y}}_{driving miles} = {\widehat{\beta }}_{intercept} + {\widehat{\beta }}_{ADI}{BG}_{ADI},$$ where $${\widehat{Y}}_{driving miles}$$ is the log-transformed driving distance to the nearest FP, $${\beta }_{intercept}$$ is the coefficient estimate for the intercept of the regression model, and $${\beta }_{ADI}$$ is the mean estimates of the ADI of BGs ($${BG}_{ADI})$$.

Although we use population center, BGs can still vary widely in geographic extent, especially in rural areas, so we included land area to adjust for any remaining spatial effects, yielding *Model* 3 (urban): ${\widehat{Y}}_{travel time} ={\widehat{\beta }}_{intercept} +{\widehat{\beta }}_{ADI} {BG}_{ADI}+{\widehat{\beta }}_{land area} {BG}_{land area}$ for BGs in urban areas, and *Model* 4 (rural): ${\widehat{Y}}_{driving miles} = {\widehat{\beta }}_{intercept} + {\widehat{\beta }}_{ADI}{BG}_{ADI}+ {\widehat{\beta }}_{land area} {BG}_{land area}$ for BGs in rural areas where ${\widehat{\beta }}_{land area}$ is the mean estimate of the land area of BGs (${BG}_{land area}$). Each covariate was tested for statistical significance with the z-test.

To assess disparities in FP accessibility by demographic and socioeconomic characteristics, we fit separate generalized linear regression models for each variable of interest, including age, gender, race/ethnicity, education, income, labor force status, poverty status, public assistance, and household composition. Each variable was modeled separately in urban and rural settings. Most predictors were categorized into quartiles, with the lowest quartile (Q1) serving as the reference group. For variables with sparse or skewed distributions, we used tertiles or binary classifications (zero vs. nonzero) instead. For example, racial/ethniccomposition variables, originally continuous values representing the proportion of each racial/ethnic group within a BG, were recoded based on their distributions. Non-Hispanic White and Hispanic/Latino populations were categorized into quartiles, while non-Hispanic Other was divided into tertiles due to limited spread. Variables with a high proportion of zeros, such as non-Hispanic Asian and Native American populations, were categorized as zero vs. nonzero presence, with zero serving as the reference. The general form of these models was: ${\widehat{Y}}_{travel time} ={\widehat{\beta }}_{intercept}+ \sum_{k=2}^{K}{\beta }_{k}{D}_{k}$ for urban areas and ${\widehat{Y}}_{travel distance} ={\widehat{\beta }}_{intercept}+ \sum_{k=2}^{K}{\beta }_{k}{D}_{k}$ for rural areas. Here ${\widehat{Y}}_{travel time}$ and ${\widehat{Y}}_{travel distance}$ represent log-transformed travel time (urban) or driving distance (rural) to the nearest FP, ${\beta }_{intercept}$ is the coefficient estimates of the intercept of the regression model, ${D}_{k}$ are dummy indicators for the higher quantiles (Q2–Q4 or T2–T3).

We also included land area and ADI as additional covariates in adjusted models: ${\widehat{Y}}_{travel time} ={\widehat{\beta }}_{intercept}+ \sum_{k=2}^{K}{\beta }_{k}{D}_{k}+ {\widehat{\beta }}_{ADI}{BG}_{ADI}+ {\widehat{\beta }}_{land area} {BG}_{land area}$ for urban areas and ${\widehat{Y}}_{travel distance} ={\widehat{\beta }}_{intercept}+ \sum_{k=2}^{K}{\beta }_{k}{D}_{k}+ {\widehat{\beta }}_{ADI}{BG}_{ADI}+ {\widehat{\beta }}_{land area} {BG}_{land area}$ for rural areas. All models were estimated independently by urban/rural areas. Each covariate was tested for statistical significance with the z-test.

### Software usage

We conducted all data analyses and statistical modeling in Python 3.8, Pandas 2.0, SciPy 1.10, NumPy 1.24, and statsmodels 0.14.0, and created bar plots in Seaborn 0.12 and Matplotlib 3.7. We created U.S. maps using Tableau Desktop 2023.1 and Python 3.12. All related datasets and codes will be publicly available after acceptance.

## Supplementary Information

Below is the link to the electronic supplementary material.

Supplementary Material 1

## Data Availability

The datasets used and/or analyzed during the current study are available from the corresponding author on reasonable request. In addition, the datasets generated and/or analyzed during the current study will be made publicly available at https://osf.io/px9t8/ upon acceptance of this manuscript for publication.
