# Supplementary material for: Socioeconomic, demographic and geographic disparities in accessibility to food pantries in the united States
Source: Sci Rep. 2026 Jan 26;16:6248. doi: 10.1038/s41598-026-35784-z (PMC12905298; doi:10.1038/s41598-026-35784-z)
Supplement: Supplementary file 1 — Supplementary Material 1 [file 41598_2026_35784_MOESM1_ESM.docx]

**Supplementary Information**

Last Updated: Dec 24^th^, 2025

**[1] Supplementary Figure 1**


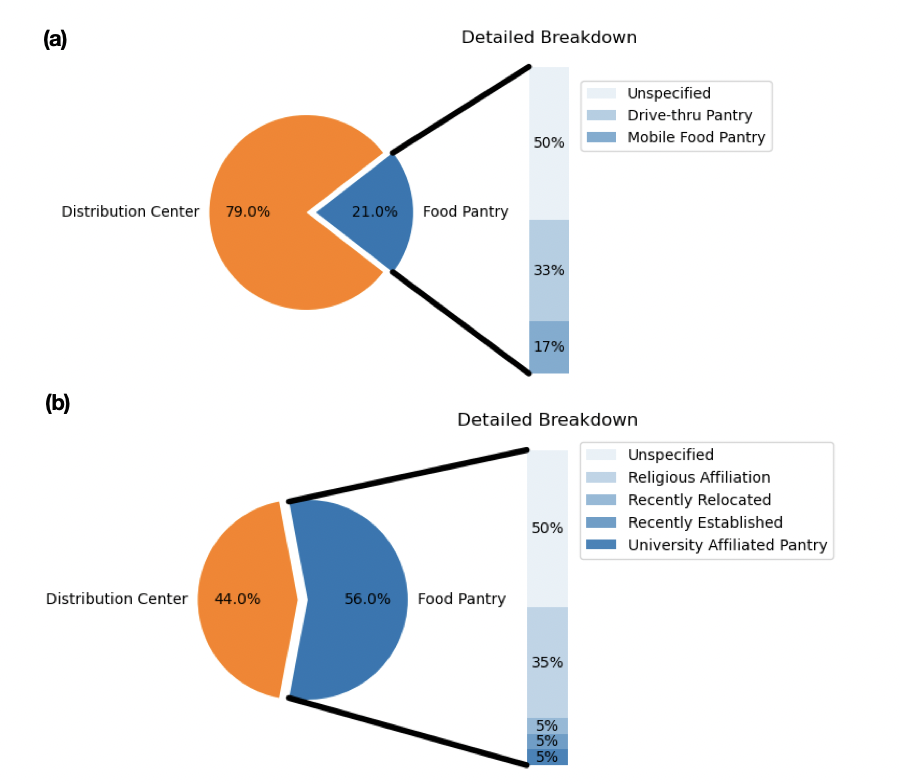


**Supplementary Figure 1.** The distribution of missing FPs in the **(a)** first dataset and **(b)** second dataset.

**[2] Supplementary Figure 2**

**(a)**


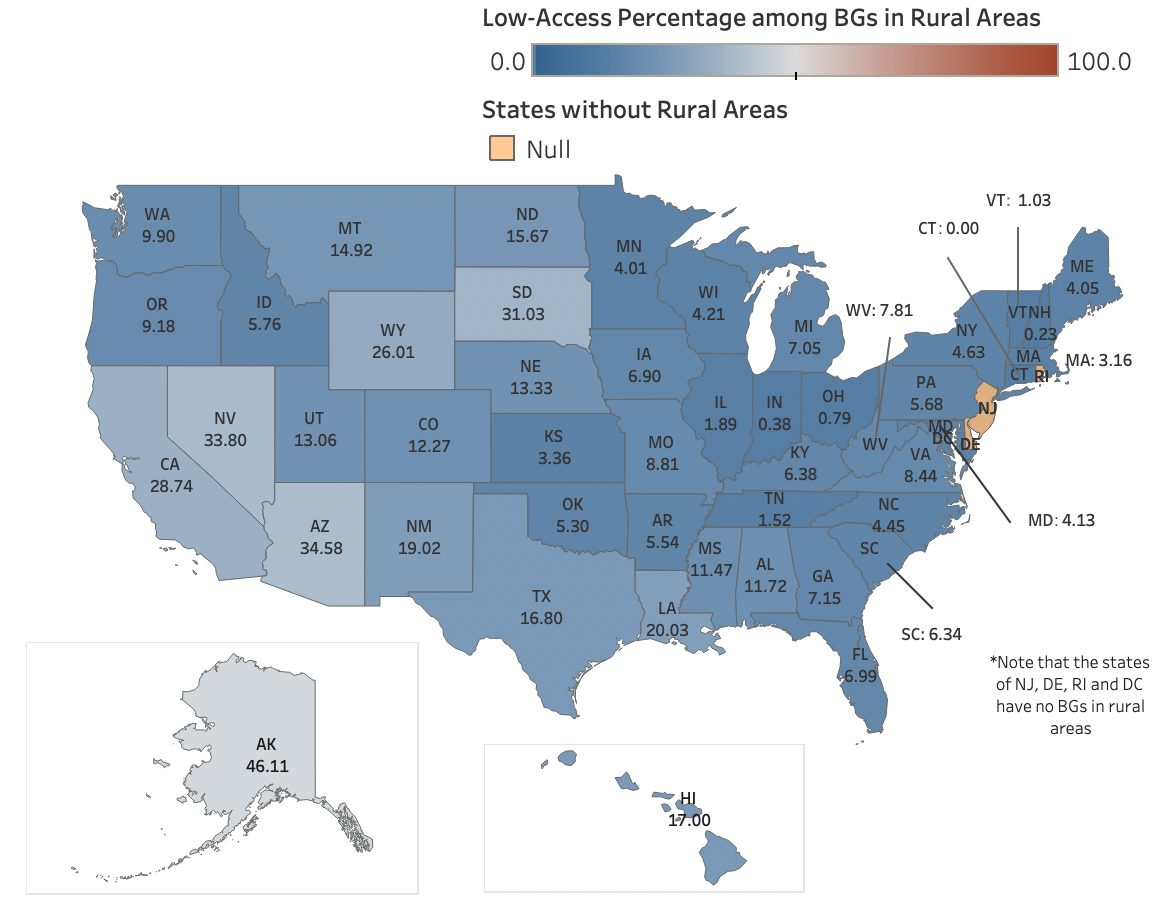


**(b)**


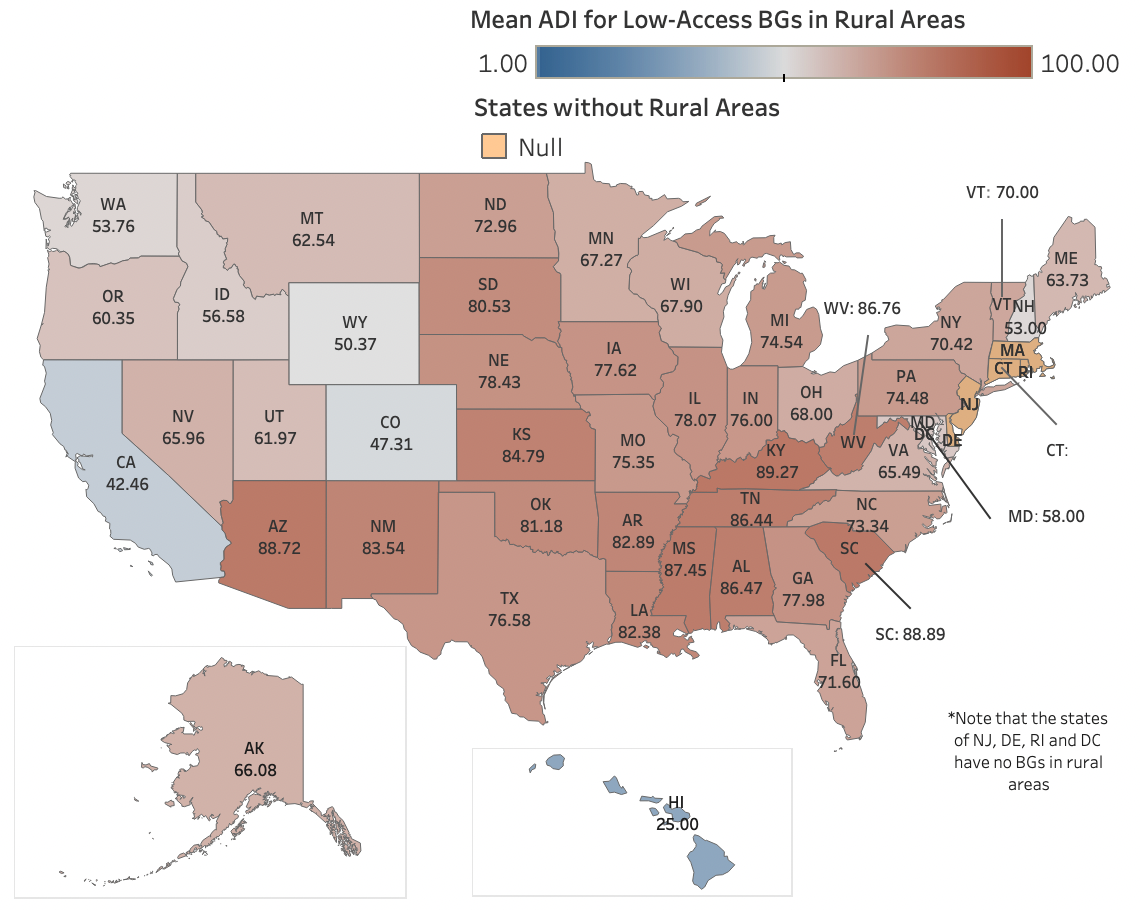


Supplementary Figure 2. (a) Percentages of BGs in rural areas with low access to FPs, by state; (b) mean ADI of low access BGs in rural areas, by state. States of NJ, DE, RI and DC have no BGs in rural areas are colored yellow. (Map generated by Tableau Desktop 2023.2 https://www.tableau.com/)

**[3] Supplementary Figure 3**

**(a)**


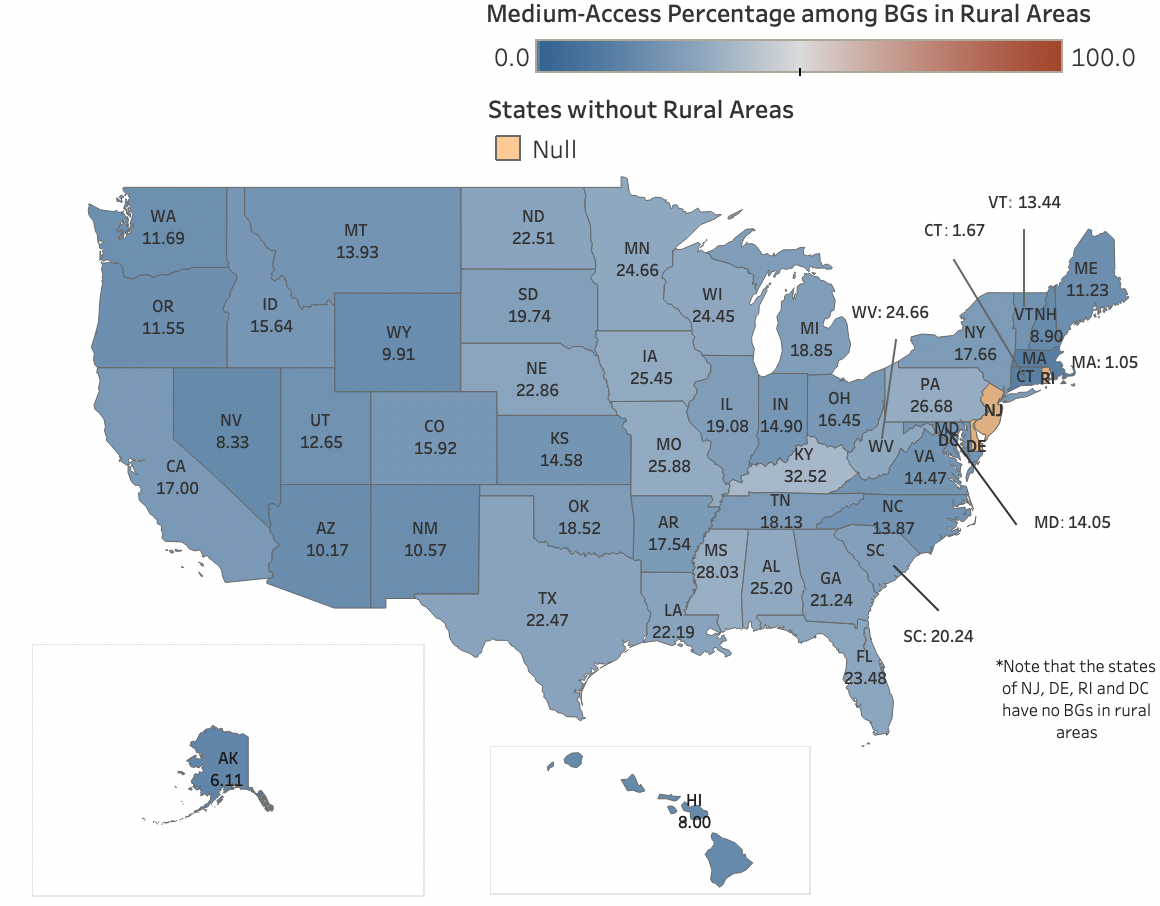


**(b)**


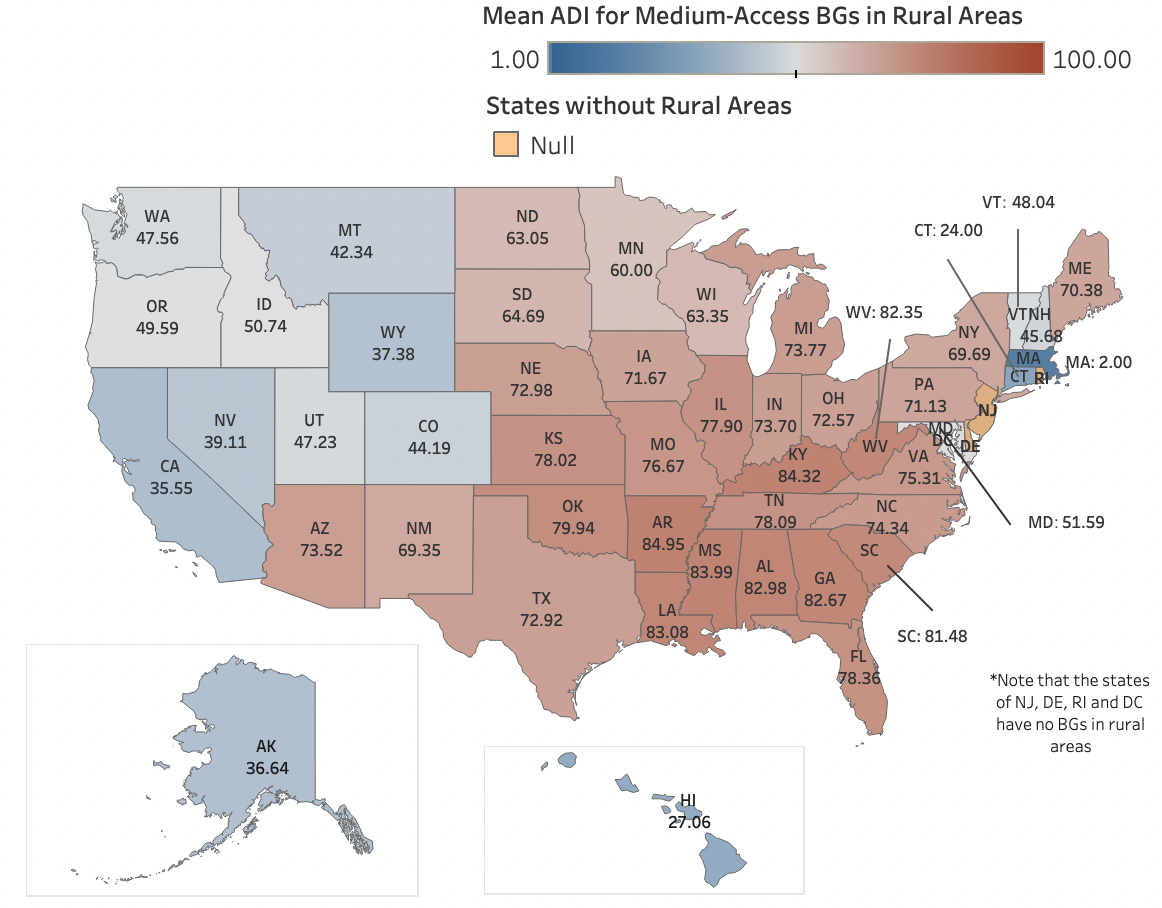


**Supplementary Figure 3.** (a) Percentages of BGs in rural areas with medium access to FPs, by state; (b) mean ADI of medium access BGs in rural areas, by state. States of NJ, DE, RI and DC have no BGs in rural areas are colored yellow. (Map generated by Tableau Desktop 2023.2 https://www.tableau.com/)

**[4] Supplementary Figure 4**

**(a)**


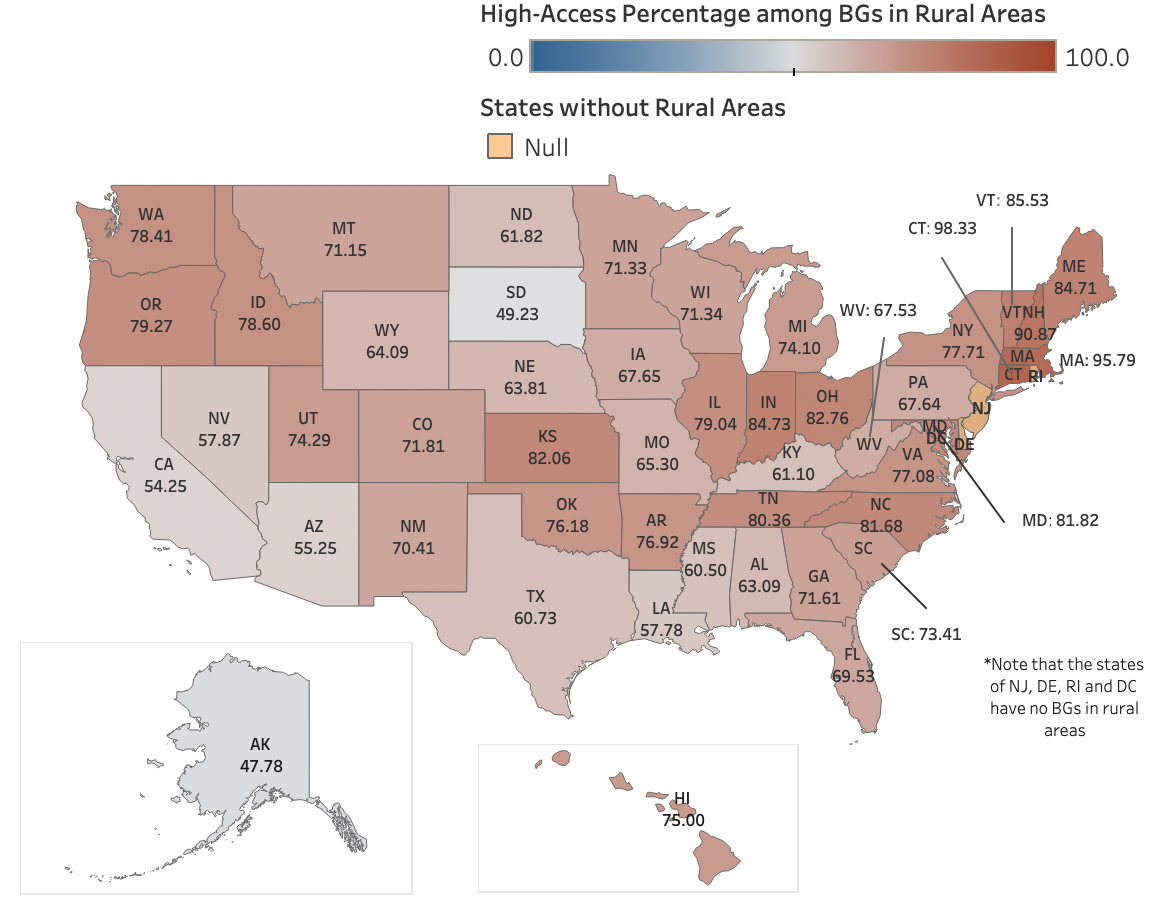


**(b)**


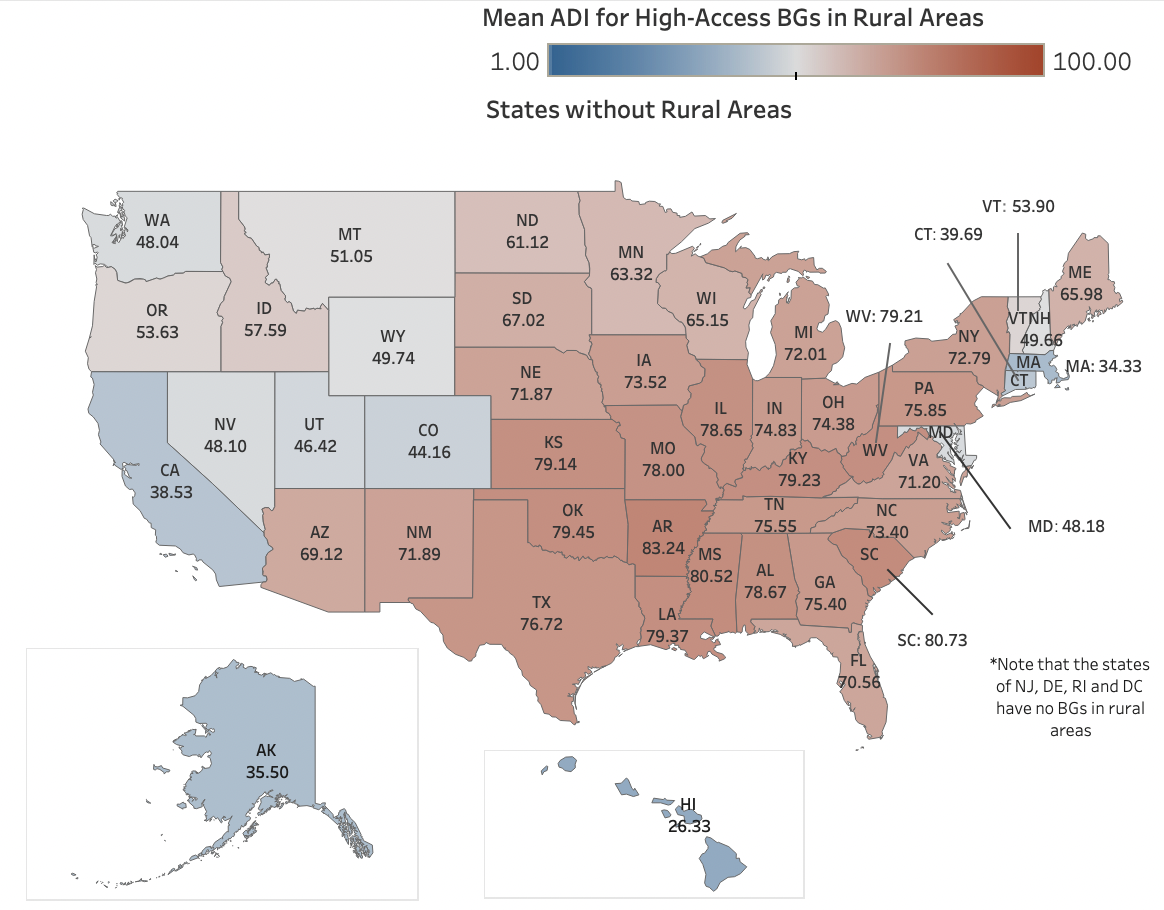


**Supplementary Figure 4.** (a) Percentages of BGs in rural areas with high access to FPs, by state; (b) mean ADI of high access BGs in rural areas, by state. States of NJ, DE, RI and DC have no BGs in rural areas are colored yellow. (Map generated by Tableau Desktop 2023.2 https://www.tableau.com/)

**[5] Supplementary Figure 5**

**(a)**


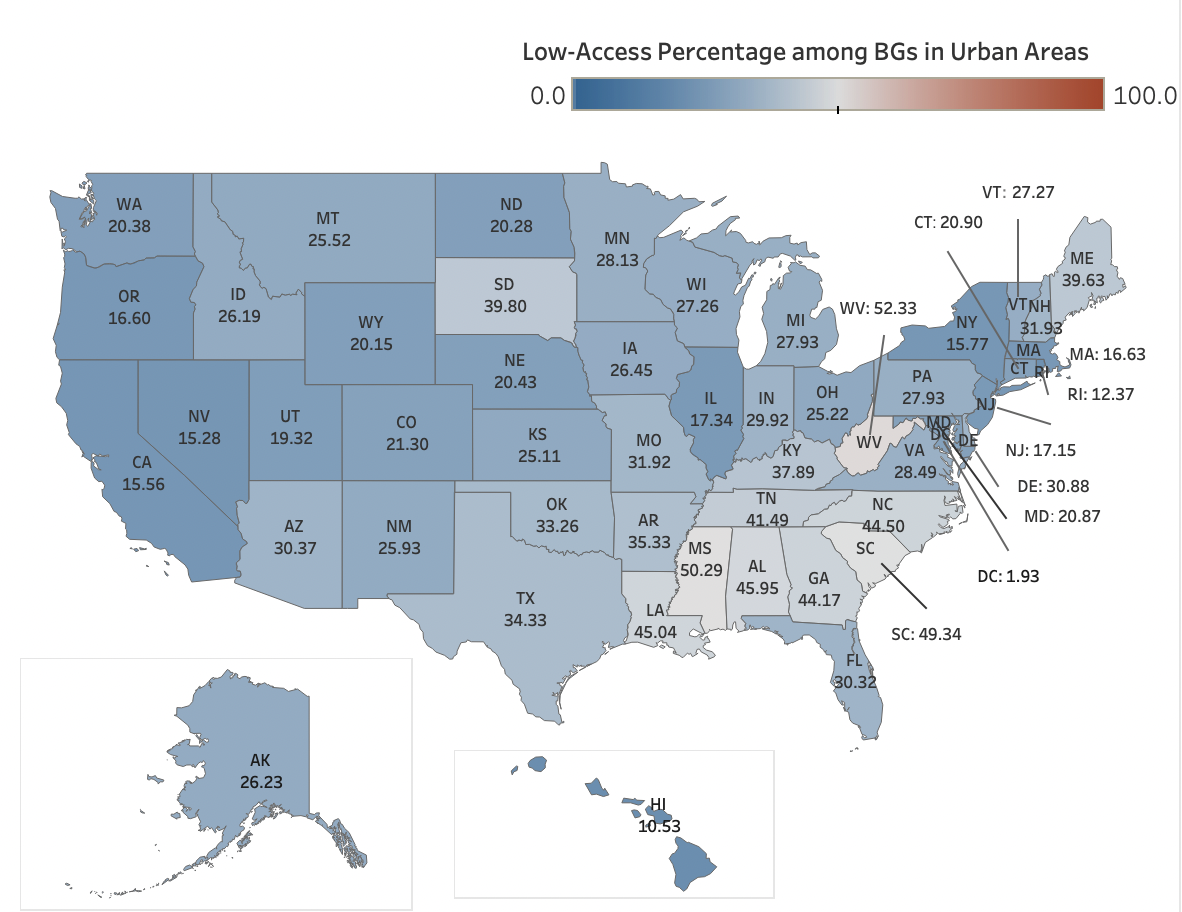


**(b)**


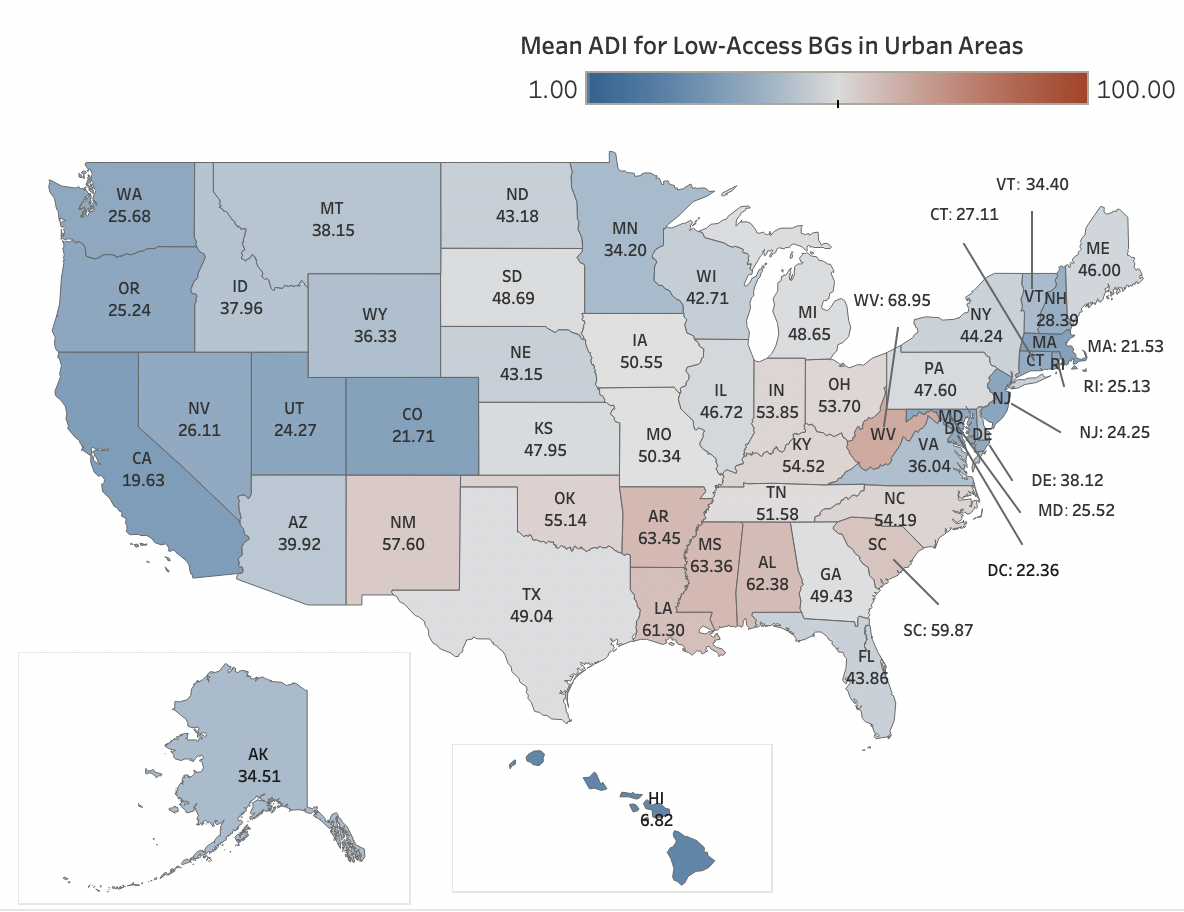


**Supplementary Figure 5.** (a) Percentages of BGs in urban areas with low access to FPs, by state; (b) mean ADI of low access BGs in urban areas, by state. (Map generated by Tableau Desktop 2023.2 https://www.tableau.com/)

**[6] Supplementary Figure 6**

**(a)**


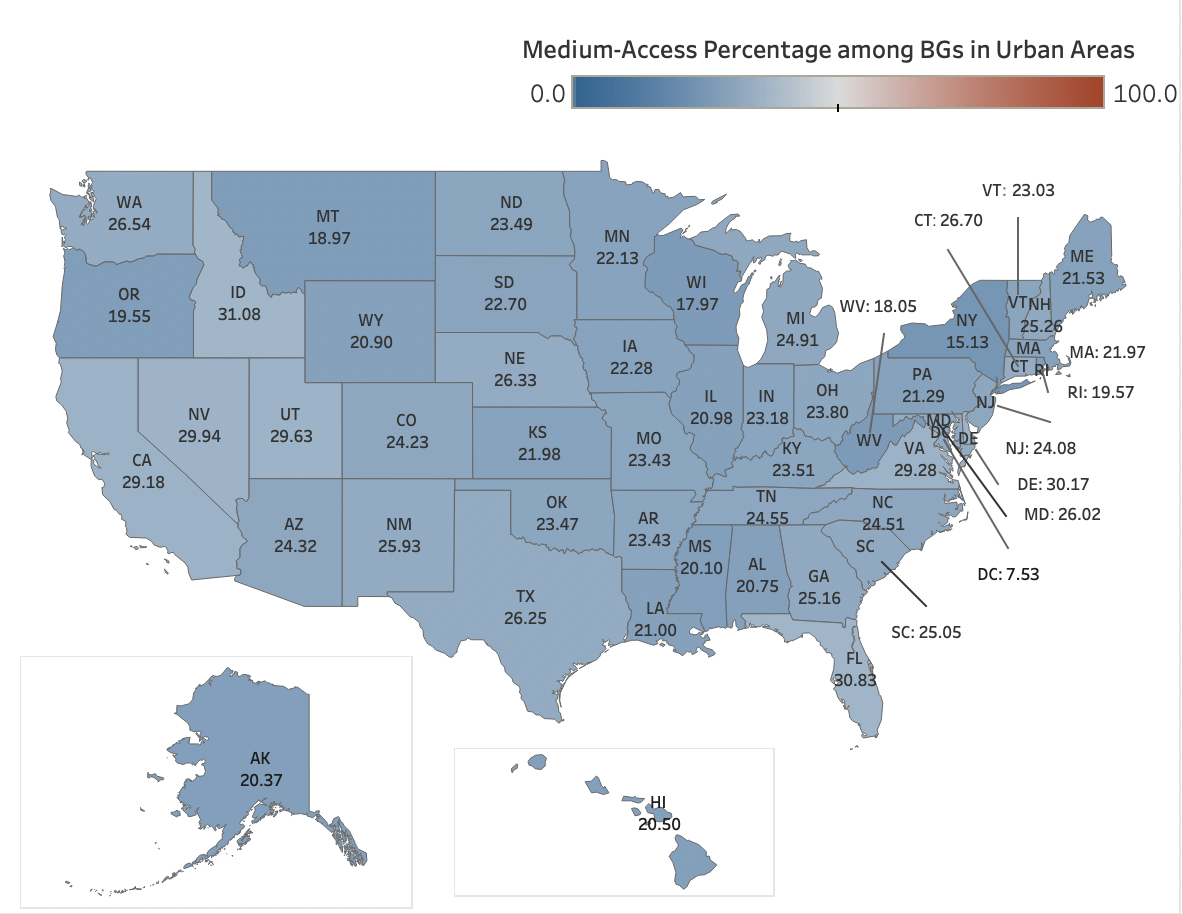


**(b)**


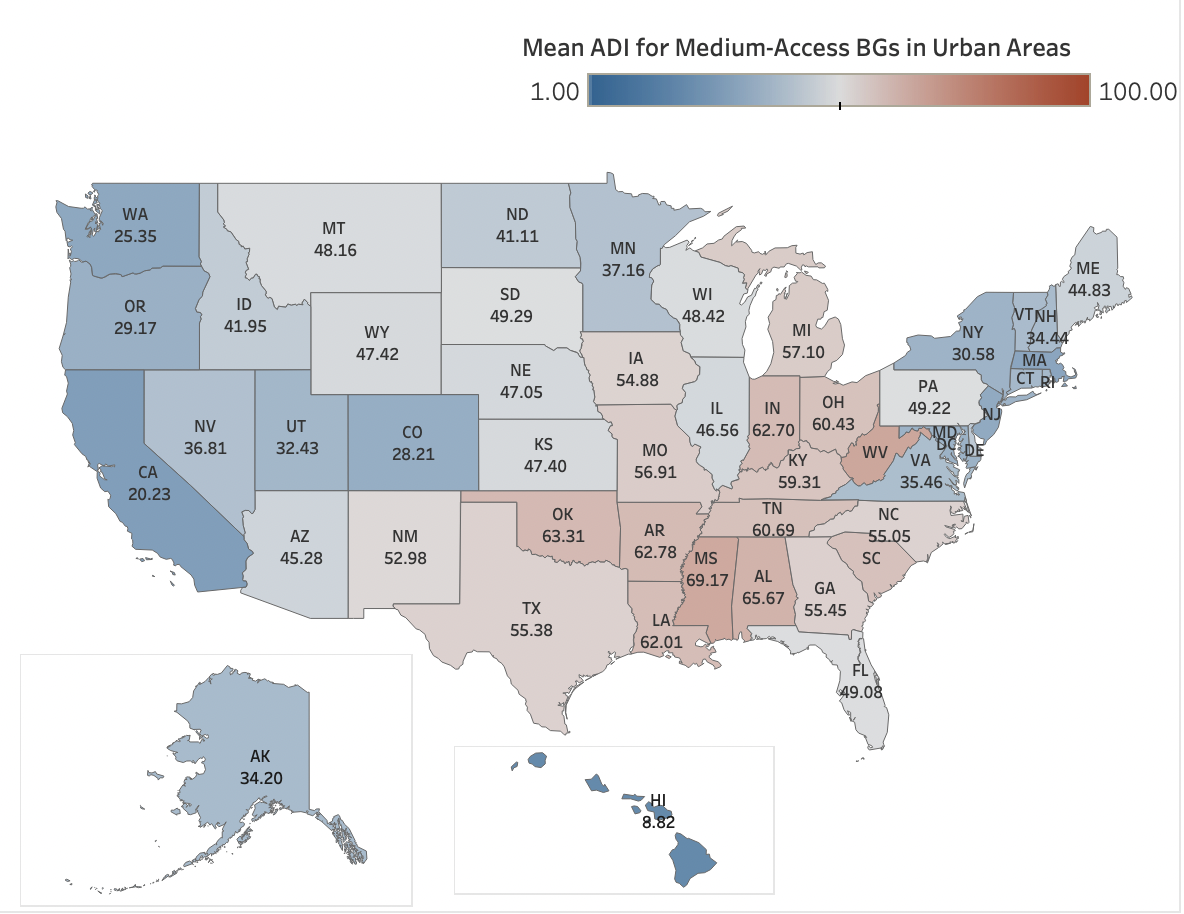


**Supplementary Figure 6.** (a) Percentages of BGs in urban areas with medium access to FPs, by state; (b) mean ADI of medium access BGs in urban areas, by state. (Map generated by Tableau Desktop 2023.2 https://www.tableau.com/)

**[7] Supplementary Figure 7**

**(a)**


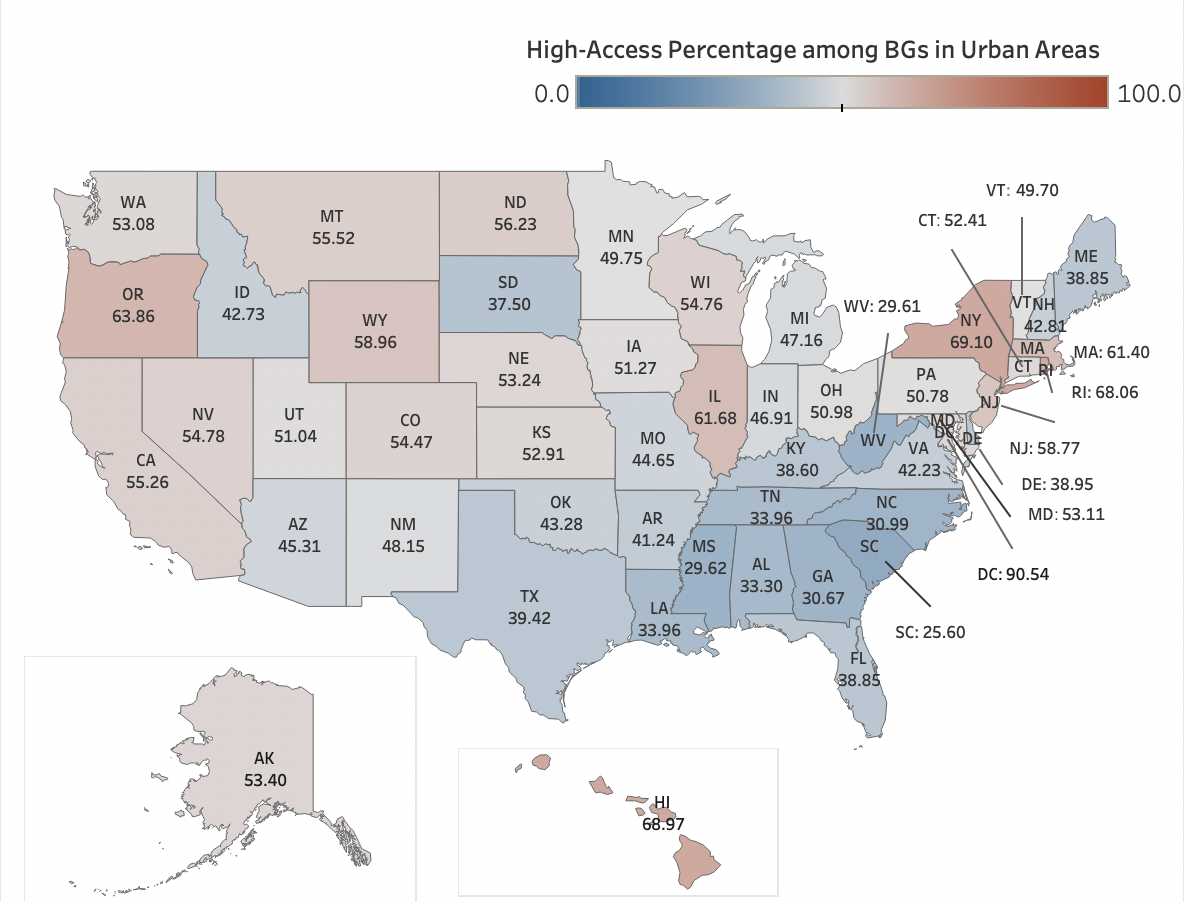


**(b)**


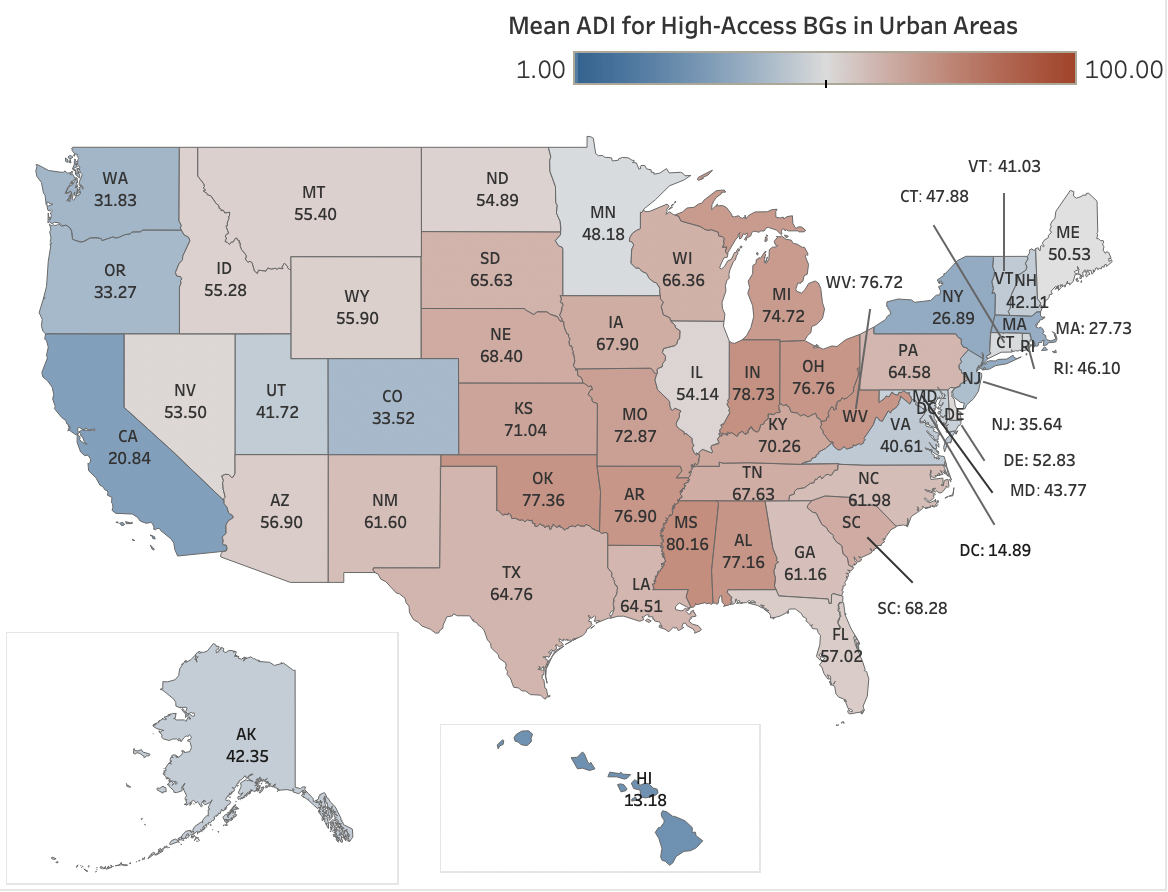


**Supplementary Figure 7.** (a) Percentages of BGs in urban areas with high access to FPs, by state; (b) mean ADI of high access BGs in urban areas, by state. (Map generated by Tableau Desktop 2023.2 https://www.tableau.com/)

**[8] Supplementary Figure 8**


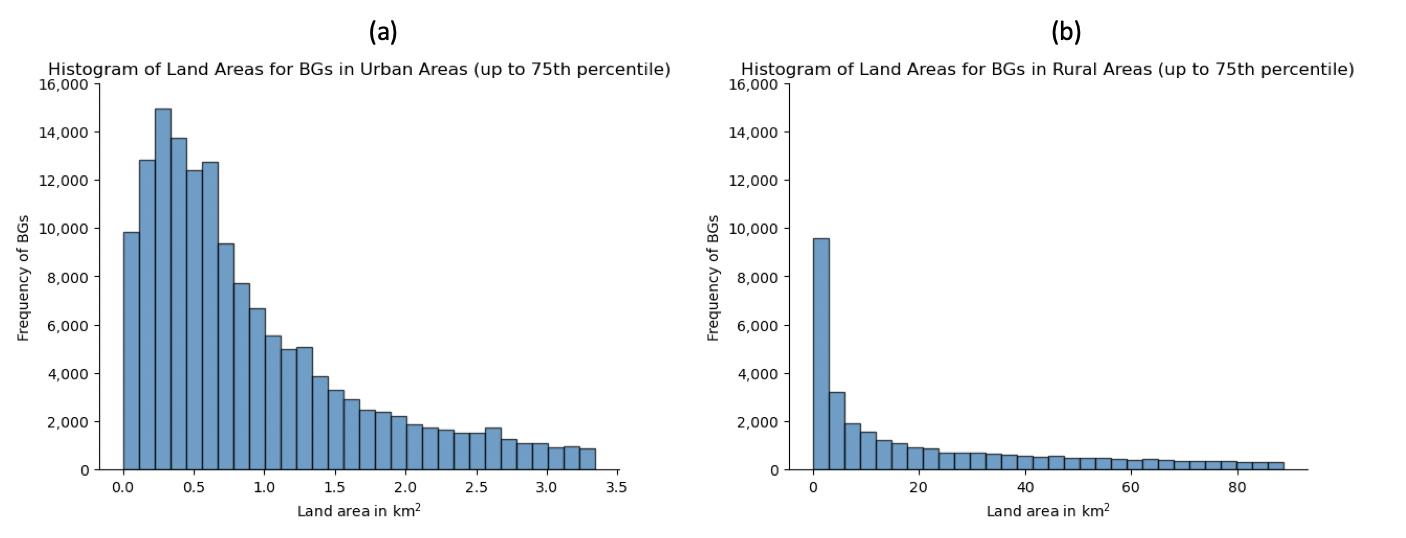


**Supplementary Figure 8.** Histograms of land areas (in km^2^) for BGs in **(a)** urban and **(b)** rural areas.

**[9] Supplementary Table 1. Results of four generalized linear regression models measuring the association between ADI and log-transformed accessibility to FPs.**

|  | **Model 1: Urban Areas**  ($\beta$, 95% Confidence Interval) | **Model 2: Rural Areas**  ($\beta$, 95% Confidence Interval) |
| --- | --- | --- |
| ADI | -0.0016***  [-0.002, -0.001] | −0.0039***  [−0.004, -0.003] |
| Constant | 3.59***  [3.58, 3.60] | 1.98***  [1.95, 2.02] |

|  | **Model 3: Urban Areas**  ($\beta$, 95% Confidence Interval) | **Model 4: Rural Areas**  ($\beta$, 95% Confidence Interval) |
| --- | --- | --- |
| ADI | -0.0017***  [-0.002, -0.002] | -0.0032***  [−0.004, -0.003] |
| Land Area | 1.18×10^-9^***  [1.15×10^-9^, 1.22×10^-9^] | 5.73×10^-10^***  [5.50×10^-10^, 5.96×10^-10^] |
| Constant | 3.58***  [3.58, 3.59] | 1.87***  [1.83, 1.90] |

**Model 1** comprises a single variable (ADI) for each BG in urban areas. **Model 2** comprises a single variable (ADI) for each BG in rural areas. **Model 3** comprises two variables (ADI and land area) for each BG in urban areas. **Model 4** comprises two variables (ADI and land area) for each BG in rural areas. *** p < 0.001, ** p < 0.01, * p < 0.05.

**[10] Supplementary Table 2. Regression estimates of the association between BG age composition and log-transformed travel time or distance to the nearest FP in (a) urban and (b) rural areas in the U.S.**

**2 (a)**

|  |  | Unadjusted | | | | Adjusted for ADI, land area | | |
| --- | --- | --- | --- | --- | --- | --- | --- | --- |
|  |  | Estimate | Standard Error | | p-value | Estimate | Standard Error | p-value |
| Age |  |  |  |  | |  |  |  |
| 18–<30 | Q4^a^ | -0.60 | 0.006 | <0.001 | | -0.58 | 0.006 | <0.001 |
|  | Q3^a^ | -0.33 | 0.006 | <0.001 | | -0.32 | 0.006 | <0.001 |
|  | Q2^a^ | -0.12 | 0.006 | <0.001 | | -0.12 | 0.006 | <0.001 |
|  | Q1^a^ | *ref* |  |  | |  |  |  |
| 30–<40 | Q4 | -0.42 | 0.006 | <0.001 | | -0.43 | 0.006 | <0.001 |
|  | Q3 | -0.26 | 0.006 | <0.001 | | -0.26 | 0.006 | <0.001 |
|  | Q2 | -0.11 | 0.006 | <0.001 | | -0.12 | 0.006 | <0.001 |
|  | Q1 | *ref* |  |  | |  |  |  |
| 40–<50 | Q4 | 0.13 | 0.006 | <0.001 | | 0.11 | 0.006 | <0.001 |
|  | Q3 | 0.10 | 0.006 | <0.001 | | 0.08 | 0.006 | <0.001 |
|  | Q2 | 0.08 | 0.006 | <0.001 | | 0.06 | 0.006 | <0.001 |
|  | Q1 | *ref* |  |  | |  |  |  |
| 50–<60 | Q4 | 0.40 | 0.006 | <0.001 | | 0.38 | 0.006 | <0.001 |
|  | Q3 | 0.29 | 0.006 | <0.001 | | 0.28 | 0.006 | <0.001 |
|  | Q2 | 0.16 | 0.006 | <0.001 | | 0.15 | 0.006 | <0.001 |
|  | Q1 | *ref* |  |  | |  |  |  |
| 60–<65 | Q4 | 0.35 | 0.006 | <0.001 | | 0.33 | 0.006 | <0.001 |
|  | Q3 | 0.26 | 0.006 | <0.001 | | 0.25 | 0.006 | <0.001 |
|  | Q2 | 0.15 | 0.006 | <0.001 | | 0.14 | 0.006 | <0.001 |
|  | Q1 | *ref* |  |  | |  |  |  |
| 65 and older | Q4 | 0.45 | 0.006 | <0.001 | | 0.44 | 0.006 | <0.001 |
|  | Q3 | 0.35 | 0.006 | <0.001 | | 0.34 | 0.006 | <0.001 |
|  | Q2 | 0.18 | 0.006 | <0.001 | | 0.18 | 0.006 | <0.001 |
|  | Q1 | *ref* |  |  | |  |  |  |

**2 (b)**

|  |  | Unadjusted | | | Adjusted for ADI, land area | | |
| --- | --- | --- | --- | --- | --- | --- | --- |
|  |  | Estimate | Standard Error | p-value | Estimate | Standard Error | p-value |
| Age |  |  |  |  |  |  |  |
| 18–<30 | Q4^a^ | -0.45 | 0.013 | <0.001 | -0.38 | 0.013 | <0.001 |
|  | Q3^a^ | -0.14 | 0.013 | <0.001 | -0.11 | 0.013 | <0.001 |
|  | Q2^a^ | -0.05 | 0.013 | <0.001 | -0.04 | 0.013 | <0.01 |
|  | Q1^a^ | *ref* |  |  |  |  |  |
| 30–<40 | Q4 | -0.27 | 0.013 | <0.001 | -0.26 | 0.013 | <0.001 |
|  | Q3 | -0.09 | 0.013 | <0.001 | -0.09 | 0.013 | <0.001 |
|  | Q2 | -0.02 | 0.013 | <0.001 | -0.04 | 0.013 | <0.01 |
|  | Q1 | *ref* |  |  |  |  |  |
| 40–<50 | Q4 | 0.04 | 0.013 | <0.001 | 0.03 | 0.013 | <0.1 |
|  | Q3 | 0.11 | 0.013 | <0.001 | 0.10 | 0.013 | <0.001 |
|  | Q2 | 0.11 | 0.006 | <0.01 | 0.09 | 0.013 | <0.001 |
|  | Q1 | *ref* |  |  |  | 0.013 |  |
| 50–<60 | Q4 | 0.31 | 0.013 | <0.001 | 0.28 | 0.013 | <0.001 |
|  | Q3 | 0.29 | 0.013 | <0.001 | 0.25 | 0.013 | <0.001 |
|  | Q2 | 0.19 | 0.013 | <0.001 | 0.16 | 0.013 | <0.001 |
|  | Q1 | *ref* |  |  |  |  |  |
| 60–<65 | Q4 | 0.32 | 0.013 | <0.001 | 0.26 | 0.013 | <0.001 |
|  | Q3 | 0.29 | 0.013 | <0.001 | 0.25 | 0.013 | <0.001 |
|  | Q2 | 0.19 | 0.013 | <0.001 | 0.17 | 0.013 | <0.001 |
|  | Q1 | *ref* |  |  |  |  |  |
| 65 and older | Q4 | 0.36 | 0.013 | <0.001 | 0.31 | 0.013 | <0.001 |
|  | Q3 | 0.34 | 0.013 | <0.001 | 0.31 | 0.013 | <0.001 |
|  | Q2 | 0.25 | 0.013 | <0.001 | 0.24 | 0.013 | <0.001 |
|  | Q1 | *ref* |  |  |  |  |  |

^a^Q1–Q4 represent quartiles of the percentage of residents in a given age group within each census block group

Q1 = lowest quartile (reference category), Q2 = second quartile, Q3 = third quartile, Q4 = highest quartile

**[11] Supplementary Table 3. Regression estimates of the association between BG gender composition and log-transformed travel time or distance to the nearest FP in (a) urban and (b) rural areas in the U.S.**

**3 (a)**

|  |  | Unadjusted | | | Adjusted for ADI, land area | | |
| --- | --- | --- | --- | --- | --- | --- | --- |
|  |  | Estimate | Standard Error | p-value | Estimate | Standard Error | p-value |
| Gender |  |  |  |  |  |  |  |
| Male | Q4^a^ | 0.14 | 0.006 | <0.001 | 0.12 | 0.006 | <0.001 |
|  | Q3^a^ | 0.32 | 0.006 | <0.001 | 0.30 | 0.006 | <0.001 |
|  | Q2^a^ | 0.24 | 0.006 | <0.001 | 0.22 | 0.006 | <0.001 |
|  | Q1^a^ | *ref* |  |  |  |  |  |
| Female | Q4 | -0.15 | 0.006 | <0.001 | -0.12 | 0.006 | <0.001 |
|  | Q3 | 0.09 | 0.006 | <0.001 | 0.10 | 0.006 | <0.001 |
|  | Q2 | 0.18 | 0.006 | <0.001 | 0.18 | 0.006 | <0.001 |
|  | Q1 | *ref* |  |  |  |  |  |

**3 (b)**

|  |  | Unadjusted | | | Adjusted for ADI, land area | | |
| --- | --- | --- | --- | --- | --- | --- | --- |
|  |  | Estimate | Standard Error | p-value | Estimate | Standard Error | p-value |
| Gender |  |  |  |  |  |  |  |
| Male | Q4^a^ | 0.27 | 0.013 | <0.001 | 0.20 | 0.013 | <0.001 |
|  | Q3^a^ | 0.34 | 0.013 | <0.001 | 0.28 | 0.013 | <0.001 |
|  | Q2^a^ | 0.22 | 0.013 | <0.001 | 0.18 | 0.013 | <0.001 |
|  | Q1^a^ | *ref* |  |  |  |  |  |
| Female | Q4 | -0.28 | 0.013 | <0.001 | -0.21 | 0.013 | <0.001 |
|  | Q3 | -0.05 | 0.013 | <0.001 | -0.02 | 0.013 | 0.11 |
|  | Q2 | 0.06 | 0.013 | <0.001 | 0.07 | 0.013 | <0.001 |
|  | Q1 | *ref* |  |  |  |  |  |

^a^Q1–Q4 represent quartiles of the percentage of residents in a given gender group within each census block group

Q1 = lowest quartile (reference category), Q2 = second quartile, Q3 = third quartile, Q4 = highest quartile

**[12] Supplementary Table 4. Regression estimates of the association between BG racial and ethnic composition and log-transformed travel time or distance to the nearest FP in (a) urban and (b) rural areas in the U.S.**

**4 (a)**

|  |  | Unadjusted | | | Adjusted for ADI, land area | | |
| --- | --- | --- | --- | --- | --- | --- | --- |
|  |  | Estimate | Standard Error | p-value | Estimate | Standard Error | p-value |
| Race/Ethnicity |  |  |  |  |  |  |  |
| Non-Hispanic White | Q4^a^ | 0.92 | 0.006 | <0.001 | 0.90 | 0.006 | <0.001 |
|  | Q3^a^ | 0.54 | 0.006 | <0.001 | 0.53 | 0.006 | <0.001 |
|  | Q2^a^ | 0.33 | 0.006 | <0.001 | 0.32 | 0.006 | <0.001 |
|  | Q1^a^ | *ref* |  |  |  |  |  |
| Non-Hispanic Black | T3^b^ | -0.52 | 0.005 | <0.001 | -0.51 | 0.006 | <0.001 |
|  | T2^b^ | -0.24 | 0.005 | <0.001 | -0.22 | 0.005 | <0.001 |
|  | T1^b^ | *ref* |  |  |  |  |  |
| Non-Hispanic Native American | Nonzero | 0.08 | 0.006 | <0.001 | 0.07 | 0.006 | <0.001 |
|  | Zero | *ref* |  |  |  |  |  |
| Non-Hispanic Asian | Nonzero | -0.15 | 0.005 | <0.001 | -0.20 | 0.005 | <0.001 |
|  | Zero | *ref* |  |  |  |  |  |
| Non-Hispanic Other | T3 | -0.12 | 0.006 | <0.001 | -0.12 | 0.006 | <0.001 |
|  | T2 | 0.02 | 0.006 | <0.001 | 0.01 | 0.006 | <0.1 |
|  | T1 | *ref* |  |  |  |  |  |
| Hispanic/Latino | Q4 | -0.56 | 0.006 | <0.001 | -0.56 | 0.006 | <0.001 |
|  | Q3 | -0.33 | 0.006 | <0.001 | -0.35 | 0.006 | <0.001 |
|  | Q2 | -0.16 | 0.006 | <0.001 | -0.17 | 0.006 | <0.001 |
|  | Q1 | *ref* |  |  |  |  |  |

**4 (b)**

|  |  | Unadjusted | | | Adjusted for ADI, land area | | |
| --- | --- | --- | --- | --- | --- | --- | --- |
|  |  | Estimate | Standard Error | p-value | Estimate | Standard Error | p-value |
| Race/Ethnicity |  |  |  |  |  |  |  |
| Non-Hispanic White | Q4^a^ | 0.39 | 0.013 | <0.001 | 0.37 | 0.013 | <0.01 |
|  | Q3^a^ | 0.21 | 0.013 | <0.001 | 0.18 | 0.013 | <0.001 |
|  | Q2^a^ | 0.06 | 0.013 | <0.001 | 0.04 | 0.013 | <0.001 |
|  | Q1^a^ | *ref* |  |  |  |  |  |
| Non-Hispanic Black | Nonzero | -0.20 | 0.009 | <0.001 | -0.16 | 0.009 | <0.001 |
|  | Zero | *ref* |  |  |  |  |  |
| Non-Hispanic Native American | Nonzero | 0.05 | 0.011 | <0.001 | -0.01 | 0.010 | 0.22 |
|  | Zero | *ref* |  |  |  |  |  |
| Non-Hispanic Asian | Nonzero | -0.08 | 0.010 | <0.001 | -0.11 | 0.010 | <0.001 |
|  | Zero | *ref* |  |  |  |  |  |
| Non-Hispanic Other | T3^b^ | -0.11 | 0.011 | <0.001 | -0.13 | 0.011 | <0.001 |
|  | T2^b^ | 0.07 | 0.011 | <0.001 | 0.04 | 0.011 | <0.001 |
|  | T1^b^ | *ref* |  |  |  |  |  |
| Hispanic/Latino | T3 | -0.29 | 0.011 | <0.001 | -0.32 | 0.011 | <0.001 |
|  | T2 | -0.04 | 0.011 | <0.001 | -0.07 | 0.011 | <0.001 |
|  | T1 | *ref* |  |  |  |  |  |

^a^Q1–Q4 represent quartiles of the percentage of residents in a given race/ethnic group within each census block group

Q1 = lowest quartile (reference category), Q2 = second quartile, Q3 = third quartile, Q4 = highest quartile

^b^T1–T3 represent tertiles of the percentage of residents in a given racial/ethnic group within each census block group

T1 = lowest tertile (reference category), T2 = second tertile, T3 = highest tertile

**[13] Supplementary Table 5. Regression estimates of the association between BG education attainment composition and log-transformed travel time or distance to the nearest FP in (a) urban and (b) rural areas in the U.S.**

**5 (a)**

|  |  | Unadjusted | | | Adjusted for ADI, land area | | |
| --- | --- | --- | --- | --- | --- | --- | --- |
|  |  | Estimate | Standard Error | p-value | Estimate | Standard Error | p-value |
| Education attainment |  |  |  |  |  |  |  |
| Less than high school | Q4^a^ | -0.47 | 0.006 | <0.001 | -0.50 | 0.007 | <0.001 |
|  | Q3^a^ | -0.14 | 0.006 | <0.001 | -0.17 | 0.006 | <0.001 |
|  | Q2^a^ | 0.02 | 0.006 | <0.001 | -0.003 | 0.006 | <0.001 |
|  | Q1^a^ | *ref* |  |  |  |  |  |
| High school or some college | Q4 | 0.23 | 0.006 | <0.001 | 0.39 | 0.007 | <0.001 |
|  | Q3 | 0.16 | 0.006 | <0.001 | 0.27 | 0.007 | <0.001 |
|  | Q2 | 0.08 | 0.006 | <0.001 | 0.15 | 0.007 | <0.001 |
|  | Q1 | *ref* |  |  |  |  |  |
| Bachelor’s degree or higher | Q4 | 0.14 | 0.006 | <0.001 | 0.14 | 0.008 | <0.001 |
|  | Q3 | 0.24 | 0.006 | <0.001 | 0.24 | 0.007 | <0.001 |
|  | Q2 | 0.19 | 0.006 | <0.001 | 0.19 | 0.007 | <0.001 |
|  | Q1 | *ref* |  |  |  |  |  |

^a^Q1–Q4 represent quartiles of the percentage of residents in a given education attainment group within each census block group

Q1 = lowest quartile (reference category), Q2 = second quartile, Q3 = third quartile, Q4 = highest quartile

**5 (b)**

|  |  | Unadjusted | | | Adjusted for ADI, land area | | |
| --- | --- | --- | --- | --- | --- | --- | --- |
|  |  | Estimate | Standard Error | p-value | Estimate | Standard Error | p-value |
| Education attainment |  |  |  |  |  |  |  |
| Less than high school | Q4^a^ | 0.04 | 0.013 | <0.001 | 0.16 | 0.013 | <0.001 |
|  | Q3^a^ | 0.08 | 0.013 | <0.001 | 0.16 | 0.013 | <0.001 |
|  | Q2^a^ | 0.09 | 0.013 | <0.01 | 0.13 | 0.014 | <0.001 |
|  | Q1^a^ | *ref* |  |  |  |  |  |
| High school or some college | Q4 | 0.16 | 0.013 | <0.001 | 0.21 | 0.013 | <0.001 |
|  | Q3 | 0.21 | 0.013 | <0.001 | 0.24 | 0.013 | <0.001 |
|  | Q2 | 0.14 | 0.013 | <0.001 | 0.17 | 0.013 | <0.001 |
|  | Q1 | *ref* |  |  |  |  |  |
| Bachelor’s degree or higher | Q4 | -0.12 | 0.013 | <0.001 | -0.33 | 0.008 | <0.001 |
|  | Q3 | 0.05 | 0.013 | <0.001 | -0.08 | 0.007 | <0.001 |
|  | Q2 | 0.10 | 0.013 | <0.001 | 0.04 | 0.007 | <0.001 |
|  | Q1 | *ref* |  |  |  |  |  |

^a^Q1–Q4 represent quartiles of the percentage of residents in a given education attainment group within each census block group

Q1 = lowest quartile (reference category), Q2 = second quartile, Q3 = third quartile, Q4 = highest quartile

**[14] Supplementary Table 6. Regression estimates of the association between BG labor force composition and log-transformed travel time or distance to the nearest FP in (a) urban and (b) rural areas in the U.S.**

**6 (a)**

|  |  | Unadjusted | | | Adjusted for ADI, land area | | |
| --- | --- | --- | --- | --- | --- | --- | --- |
|  |  | Estimate | Standard Error | p-value | Estimate | Standard Error | p-value |

| Labor force |  |  |  |  |  |  |  |
| --- | --- | --- | --- | --- | --- | --- | --- |
| Employed | Q4^a^ | -0.10 | 0.006 | <0.001 | -0.13 | 0.007 | <0.01 |
|  | Q3^a^ | 0.03 | 0.006 | <0.001 | 0.00 | 0.007 | 0.96 |
|  | Q2^a^ | 0.04 | 0.006 | <0.001 | 0.02 | 0.006 | <0.001 |
|  | Q1^a^ | *ref* |  |  |  |  |  |
| Unemployed | Q4 | -0.37 | 0.006 | <0.001 | -0.35 | 0.006 | <0.001 |
|  | Q3 | -0.13 | 0.006 | <0.001 | -0.12 | 0.006 | <0.001 |
|  | Q2 | -0.01 | 0.006 | <0.001 | -0.001 | 0.006 | 0.88 |
|  | Q1 | *ref* |  |  |  |  |  |
| Other | Nonzero | 0.18 | 0.007 | <0.001 | 0.18 | 0.008 | <0.001 |
|  | Zero | *ref* |  |  |  |  |  |
| Not in labor force | Q4 | 0.21 | 0.006 | <0.001 | 0.24 | 0.007 | <0.001 |
|  | Q3 | 0.20 | 0.006 | <0.001 | 0.21 | 0.006 | <0.001 |
|  | Q2 | 0.15 | 0.006 | <0.001 | 0.16 | 0.006 | <0.001 |
|  | Q1 | *ref* |  |  |  |  |  |

**6 (b)**

|  |  | Unadjusted | | | Adjusted for ADI, land area | | |
| --- | --- | --- | --- | --- | --- | --- | --- |
|  |  | Estimate | Standard Error | p-value | Estimate | Standard Error | p-value |
| Labor force |  |  |  |  |  |  |  |
| Employed | Q4^a^ | -0.26 | 0.013 | <0.001 | -0.32 | 0.013 | <0.01 |
|  | Q3^a^ | -0.11 | 0.013 | <0.001 | -0.15 | 0.013 | 0.96 |
|  | Q2^a^ | -0.08 | 0.013 | <0.001 | -0.10 | 0.013 | <0.001 |
|  | Q1^a^ | *ref* |  |  |  |  |  |
| Unemployed | Q4 | -0.06 | 0.013 | <0.001 | -0.01 | 0.013 | 0.47 |
|  | Q3 | 0.07 | 0.013 | <0.001 | 0.09 | 0.013 | <0.001 |
|  | Q2 | 0.13 | 0.013 | <0.001 | 0.12 | 0.013 | 0.88 |
|  | Q1 | *ref* |  |  |  |  |  |
| Other | Nonzero | 0.00 | 0.019 | 0.94 | -0.04 | 0.019 | <0.1 |
|  | Zero | *ref* |  |  |  |  |  |
| Not in labor force | Q4 | 0.31 | 0.013 | <0.001 | 0.36 | 0.013 | <0.001 |
|  | Q3 | 0.24 | 0.013 | <0.001 | 0.27 | 0.013 | <0.001 |
|  | Q2 | 0.19 | 0.013 | <0.001 | 0.20 | 0.013 | <0.001 |
|  | Q1 | *ref* |  |  |  |  |  |

^a^Q1–Q4 represent quartiles of the percentage of residents in a given labor force group within each census block group

Q1 = lowest quartile (reference category), Q2 = second quartile, Q3 = third quartile, Q4 = highest quartile

**[15] Supplementary Table 7. Regression estimates of the association between BG yearly household income composition and log-transformed travel time or distance to the nearest FP in (a) urban and (b) rural areas in the U.S.**

**7 (a)**

|  |  | Unadjusted | | | Adjusted for ADI, land area | | |
| --- | --- | --- | --- | --- | --- | --- | --- |
|  |  | Estimate | Standard Error | p-value | Estimate | Standard Error | p-value |
| Yearly Household Income |  |  |  |  |  |  |  |
| Less than $50,000 | Q4^a^ | -0.64 | 0.006 | <0.001 | -0.97 | 0.008 | <0.001 |
|  | Q3^a^ | -0.27 | 0.006 | <0.001 | -0.48 | 0.007 | <0.001 |
|  | Q2^a^ | -0.12 | 0.006 | <0.001 | -0.22 | 0.006 | <0.001 |
|  | Q1^a^ | *ref* |  |  |  |  |  |
| $50,000 or more | Q4 | 0.64 | 0.006 | <0.001 | 0.97 | 0.008 | <0.001 |
|  | Q3 | 0.52 | 0.006 | <0.001 | 0.75 | 0.007 | <0.001 |
|  | Q2 | 0.37 | 0.006 | <0.001 | 0.50 | 0.006 | <0.001 |
|  | Q1 | *ref* |  |  |  |  |  |

**7 (b)**

|  |  | Unadjusted | | | Adjusted for ADI, land area | | |
| --- | --- | --- | --- | --- | --- | --- | --- |
|  |  | Estimate | Standard Error | p-value | Estimate | Standard Error | p-value |
| Yearly Household Income |  |  |  |  |  |  |  |
| Less than $50,000 | Q4^a^ | -0.34 | 0.013 | <0.001 | -0.30 | 0.016 | <0.001 |
|  | Q3^a^ | -0.12 | 0.013 | <0.001 | -0.11 | 0.015 | <0.001 |
|  | Q2^a^ | -0.01 | 0.013 | 0.60 | -0.01 | 0.013 | 0.56 |
|  | Q1^a^ | *ref* |  |  |  |  |  |
| $50,000 or more | Q4 | 0.34 | 0.013 | <0.001 | -0.02 | 0.016 | <0.001 |
|  | Q3 | 0.33 | 0.013 | <0.001 | -0.01 | 0.013 | <0.001 |
|  | Q2 | 0.22 | 0.013 | <0.001 | -0.01 | 0.013 | <0.001 |
|  | Q1 | *ref* |  |  |  |  |  |

^a^Q1–Q4 represent quartiles of the percentage of residents in a given yearly household income group within each census block group

Q1 = lowest quartile (reference category), Q2 = second quartile, Q3 = third quartile, Q4 = highest quartile

**[16] Supplementary Table 8. Regression estimates of the association between BG household composition and log-transformed travel time or distance to the nearest FP in (a) urban and (b) rural areas in the U.S.**

**8 (a)**

|  |  | Unadjusted | | | Adjusted for ADI, land area | | |
| --- | --- | --- | --- | --- | --- | --- | --- |
|  |  | Estimate | Standard Error | p-value | Estimate | Standard Error | p-value |
| Household Composition |  |  |  |  |  |  |  |
| Married couple with children | Q4^a^ | 0.37 | 0.006 | <0.001 | 0.37 | 0.007 | <0.001 |
|  | Q3^a^ | 0.34 | 0.006 | <0.001 | 0.33 | 0.006 | <0.001 |
|  | Q2^a^ | 0.25 | 0.006 | <0.001 | 0.24 | 0.006 | <0.001 |
|  | Q1^a^ | *ref* |  |  |  |  |  |
| Married couple without children | Q4 | 0.66 | 0.006 | <0.001 | 0.66 | 0.006 | <0.001 |
|  | Q3 | 0.54 | 0.006 | <0.001 | 0.54 | 0.006 | <0.001 |
|  | Q2 | 0.33 | 0.006 | <0.001 | 0.33 | 0.006 | <0.001 |
|  | Q1 | *ref* |  |  |  |  |  |
| Single female householder with children | T3^b^ | -0.45 | 0.005 | <0.001 | -0.46 | 0.006 | <0.001 |
|  | T2^b^ | 0.002 | 0.005 | <0.001 | -0.01 | 0.005 | <0.001 |
|  | T1^b^ | *ref* |  |  |  |  |  |
| Single female householder without children | Q4 | -0.51 | 0.006 | <0.001 | -0.51 | 0.006 | <0.001 |
|  | Q3 | -0.18 | 0.006 | <0.001 | -0.18 | 0.006 | <0.001 |
|  | Q2 | 0.06 | 0.006 | <0.001 | 0.05 | 0.006 | <0.001 |
|  | Q1 | *ref* |  |  |  |  |  |
| Single male householder with children | Nonzero | 0.06 | 0.005 | <0.001 | 0.06 | 0.005 | <0.001 |
|  | Zero | *ref* |  |  |  |  |  |
| Single male householder without children | Nonzero | -0.05 | 0.005 | <0.001 | -0.05 | 0.005 | <0.001 |
|  | Zero | *ref* |  |  |  |  |  |

**8 (b)**

|  |  | Unadjusted | | | Adjusted for ADI, land area | | |
| --- | --- | --- | --- | --- | --- | --- | --- |
|  |  | Estimate | Standard Error | p-value | Estimate | Standard Error | p-value |
| Household Composition |  |  |  |  |  |  |  |
| Married couple with children | Q4^a^ | -0.004 | 0.013 | 0.78 | -0.03 | 0.013 | <0.01 |
|  | Q3^a^ | 0.09 | 0.013 | <0.001 | 0.06 | 0.013 | <0.001 |
|  | Q2^a^ | 0.09 | 0.013 | <0.001 | 0.07 | 0.013 | <0.001 |
|  | Q1^a^ | *ref* |  |  |  |  |  |
| Married couple without children | Q4 | 0.61 | 0.013 | <0.001 | 0.55 | 0.013 | <0.001 |
|  | Q3 | 0.50 | 0.013 | <0.001 | 0.46 | 0.013 | <0.001 |
|  | Q2 | 0.32 | 0.013 | <0.001 | 0.29 | 0.013 | <0.001 |
|  | Q1 | *ref* |  |  |  |  |  |
| Single female householder with children | T3^b^ | -0.45 | 0.013 | <0.001 | -0.40 | 0.011 | <0.01 |
|  | T2^b^ | -0.04 | 0.013 | <0.001 | -0.03 | 0.011 | <0.001 |
|  | T1^b^ | *ref* |  |  |  |  |  |
| Single female householder without children | Q4 | -0.16 | 0.013 | <0.001 | -0.09 | 0.013 | <0.001 |
|  | Q3 | 0.01 | 0.013 | 0.31 | 0.04 | 0.013 | <0.01 |
|  | Q2 | 0.15 | 0.013 | <0.001 | 0.015 | 0.013 | <0.001 |
|  | Q1 | *ref* |  |  |  |  |  |
| Single male householder with children | Nonzero | 0.08 | 0.009 | <0.001 | 0.07 | 0.009 | <0.001 |
|  | Zero | *ref* |  |  |  |  |  |
| Single male householder without children | Nonzero | 0.20 | 0.009 | <0.001 | 0.19 | 0.009 | <0.001 |
|  | Zero | *ref* |  |  |  |  |  |

^a^Q1–Q4 represent quartiles of the percentage of residents in a given househole group within each census block group

Q1 = lowest quartile (reference category), Q2 = second quartile, Q3 = third quartile, Q4 = highest quartile

^b^T1–T3 represent tertiles of the percentage of residents in a given household group within each census block group

T1 = lowest tertile (reference category), T2 = second tertile, T3 = highest tertile

**[17] Supplementary Table 9. Regression estimates of the association between BG household poverty status and log-transformed travel time or distance to the nearest FP in (a) urban and (b) rural areas in the U.S.**

**9 (a)**

|  |  | Unadjusted | | | Adjusted for ADI, land area | | |
| --- | --- | --- | --- | --- | --- | --- | --- |
|  |  | Estimate | Standard Error | p-value | Estimate | Standard Error | p-value |
| Household poverty status in the past 12 months |  |  |  |  |  |  |  |
| Below poverty level | Q4^a^ | -0.67 | 0.006 | <0.001 | -0.76 | 0.007 | <0.001 |
|  | Q3^a^ | -0.28 | 0.006 | <0.001 | -0.34 | 0.007 | <0.001 |
|  | Q2^a^ | -0.08 | 0.006 | <0.001 | -0.11 | 0.006 | <0.001 |
|  | Q1^a^ | *ref* |  |  |  |  |  |
| At or above poverty level | Q4 | 0.67 | 0.006 | <0.001 | 0.76 | 0.007 | <0.001 |
|  | Q3 | 0.59 | 0.006 | <0.001 | 0.65 | 0.006 | <0.001 |
|  | Q2 | 0.39 | 0.006 | <0.001 | 0.43 | 0.006 | <0.001 |
|  | Q1 | *ref* |  |  |  |  |  |

**9 (b)**

|  |  | Unadjusted | | | Adjusted for ADI, land area | | |
| --- | --- | --- | --- | --- | --- | --- | --- |
|  |  | Estimate | Standard Error | p-value | Estimate | Standard Error | p-value |
| Household poverty status in the past 12 months |  |  |  |  |  |  |  |
| Below poverty level | Q4^a^ | -0.27 | 0.013 | <0.01 | -0.19 | 0.014 | <0.001 |
|  | Q3^a^ | -0.05 | 0.013 | <0.001 | -0.02 | 0.013 | 0.16 |
|  | Q2^a^ | 0.04 | 0.013 | <0.001 | 0.05 | 0.013 | <0.001 |
|  | Q1^a^ | *ref* |  |  |  |  |  |
| At or above poverty level | Q4 | 0.26 | 0.013 | <0.001 | 0.18 | 0.014 | <0.001 |
|  | Q3 | 0.31 | 0.013 | <0.001 | 0.24 | 0.013 | <0.001 |
|  | Q2 | 0.21 | 0.013 | <0.001 | 0.17 | 0.013 | <0.001 |
|  | Q1 | *ref* |  |  |  |  |  |

^a^Q1–Q4 represent quartiles of the percentage of residents in a given yearly household poverty status group within each census block group

Q1 = lowest quartile (reference category), Q2 = second quartile, Q3 = third quartile, Q4 = highest quartile

**[18] Supplementary Table 10. Regression estimates of the association between BG cash or SNAP assistance and log-transformed travel time or distance to the nearest FP in (a) urban and (b) rural areas in the U.S.**

**10 (a)**

|  |  | Unadjusted | | | Adjusted for ADI, land area | | |
| --- | --- | --- | --- | --- | --- | --- | --- |
|  |  | Estimate | Standard Error | p-value | Estimate | Standard Error | p-value |
| Cash public assistance income or Food Stamps/SNAP benefits in the past 12 months |  |  |  |  |  |  |  |
| With cash public assistance or Food Stamps/SNAP | Q4^a^ | -0.57 | 0.006 | <0.001 | -0.66 | 0.007 | <0.001 |
|  | Q3^a^ | -0.12 | 0.006 | <0.001 | -0.18 | 0.006 | <0.001 |
|  | Q2^a^ | 0.15 | 0.006 | <0.001 | 0.12 | 0.006 | <0.001 |
|  | Q1^a^ | *ref* |  |  |  |  |  |
| Without cash public assistance or Food Stamps/SNAP | Q4 | 0.57 | 0.006 | <0.001 | 0.66 | 0.007 | <0.001 |
|  | Q3 | 0.72 | 0.006 | <0.001 | 0.78 | 0.007 | <0.001 |
|  | Q2 | 0.45 | 0.006 | <0.001 | 0.49 | 0.006 | <0.001 |
|  | Q1 | *ref* |  |  |  |  |  |

**10 (b)**

|  |  | Unadjusted | | | Adjusted for ADI, land area | | |
| --- | --- | --- | --- | --- | --- | --- | --- |
|  |  | Estimate | Standard Error | p-value | Estimate | Standard Error | p-value |
| Cash public assistance income or Food Stamps/SNAP benefits in the past 12 months |  |  |  |  |  |  |  |
| With cash public assistance or SNAP | Q4^a^ | -0.30 | 0.013 | <0.001 | -0.23 | 0.014 | <0.001 |
|  | Q3^a^ | -0.03 | 0.013 | <0.1 | 0.02 | 0.013 | 0.23 |
|  | Q2^a^ | 0.07 | 0.013 | <0.001 | 0.08 | 0.013 | <0.001 |
|  | Q1^a^ | *ref* |  |  |  |  |  |
| Without cash public assistance or SNAP | Q4 | 0.30 | 0.013 | <0.001 | 0.22 | 0.014 | <0.001 |
|  | Q3 | 0.37 | 0.013 | <0.001 | 0.31 | 0.013 | <0.001 |
|  | Q2 | 0.27 | 0.013 | <0.001 | 0.24 | 0.013 | <0.001 |
|  | Q1 | *ref* |  |  |  |  |  |

^a^Q1–Q4 represent quartiles of the percentage of residents in a given yearly household poverty status group within each census block group

Q1 = lowest quartile (reference category), Q2 = second quartile, Q3 = third quartile, Q4 = highest quartile

**[19] Supplementary Table 11. Descriptive statistics of land area of urban and rural BGs.**

|  | **Urban BG Area in km^2^**  **(198,767 BGs)** | **Rural BG Area in km^2^**  **(41,013 BGs)** |
| --- | --- | --- |
| Mean | 12.7 | 161.0 |
| 25^th^ percentile | 0.43 | 3.46 |
| 50^th^ percentile | 0.99 | 24.5 |
| 75^th^ percentile | 3.34 | 88.8 |
| Max | 36,700 | 158,000 |

**[20] Supplementary Table 12.**

| **Variable** | **Overall** | **Low access^a^** | **Medium access^a^** | **High access^a^** |
| --- | --- | --- | --- | --- |
| Number of BGs in Rural Areas [number (proportion of total)] | 41,013 | 2,825 (6.9%) | 8,803 (21.5) | 29,385 (71.6) |
| ADI [mean (95%CI)] | 71.3 (71.1, 71.5) | 71.6 (70.7, 72.5) | 71.7 (71.3, 72.2) | 71.1 (70.8, 71.3) |
| Travel time (in minutes)  [mean (95%CI)] | 12.18 (11.9, 12.5) | 57.6 (52.7, 62.4) | 20.4 (20.3, 20.5) | 6.7 (6.6, 6.7) |

^a^Access categories for BGs in rural area were defined as high access (Nearest FP within 15-minute driving time), medium access (Nearest FP between 15–30-minute driving time) and low access (No FP within 30-minutes driving time or no FP within 25 miles)
